# Comparative Proteomic Analysis of Supportive and Unsupportive Extracellular Matrix Substrates for Human Embryonic Stem Cell Maintenance*[Fn FN2]

**DOI:** 10.1074/jbc.M113.463372

**Published:** 2013-05-08

**Authors:** Despina Soteriou, Banu Iskender, Adam Byron, Jonathan D. Humphries, Simon Borg-Bartolo, Marie-Claire Haddock, Melissa A. Baxter, David Knight, Martin J. Humphries, Susan J. Kimber

**Affiliations:** From the ‡ North West Embryonic Stem Cell Centre, Faculty of Life Sciences, University of Manchester, Manchester M13 9NT, United Kingdom,; the § Wellcome Trust Centre for Cell-Matrix Research, Faculty of Life Sciences, University of Manchester, Manchester M13 9PT, United Kingdom, and; the ¶ Biological Mass Spectrometry Core Facility, Faculty of Life Sciences, University of Manchester, Manchester M13 9PT, United Kingdom

**Keywords:** Cell Biology, Embryonic Stem Cell, Extracellular Matrix, Extracellular Matrix Proteins, Proteomics, Stem Cells

## Abstract

Human embryonic stem cells (hESCs) are pluripotent cells that have indefinite replicative potential and the ability to differentiate into derivatives of all three germ layers. hESCs are conventionally grown on mitotically inactivated mouse embryonic fibroblasts (MEFs) or feeder cells of human origin. In addition, feeder-free culture systems can be used to support hESCs, in which the adhesive substrate plays a key role in the regulation of stem cell self-renewal or differentiation. Extracellular matrix (ECM) components define the microenvironment of the niche for many types of stem cells, but their role in the maintenance of hESCs remains poorly understood. We used a proteomic approach to characterize in detail the composition and interaction networks of ECMs that support the growth of self-renewing hESCs. Whereas many ECM components were produced by supportive and unsupportive MEF and human placental stromal fibroblast feeder cells, some proteins were only expressed in supportive ECM, suggestive of a role in the maintenance of pluripotency. We show that identified candidate molecules can support attachment and self-renewal of hESCs alone (fibrillin-1) or in combination with fibronectin (perlecan, fibulin-2), in the absence of feeder cells. Together, these data highlight the importance of specific ECM interactions in the regulation of hESC phenotype and provide a resource for future studies of hESC self-renewal.

## Teaser

**Background:** Interaction of stem cells with extracellular matrix (ECM) controls their fate.

**Results:** MS reveals interacting ECM networks produced by human embryonic stem cells (hESCs) and their feeders; supportive and unsupportive hESC substrates comprise distinct ECM compositions.

**Conclusion:** Several ECM molecules maintain hESC self-renewal.

**Significance:** Better understanding of hESC self-renewal has applications in understanding development, generating cell therapies, and modeling diseases.

## Introduction

Human embryonic stem cells (hESCs)^10^ are derived from the inner cell mass of the blastocyst, and they have almost unlimited self-renewal, together with the potential to differentiate into the cell types originating from all three embryonic germ layers: endoderm, mesoderm, and ectoderm. The differentiation of embryonic stem cells *in vitro* provides a model for studying the cellular and molecular mechanisms of early development, and hESCs can be utilized as tools for drug discovery and modeling diseases (1). Although hESCs hold enormous promise for therapeutic applications, several hurdles need to be overcome before this becomes a reality (2). These include clearer definition of the factors that are required to maintain the self-renewal and pluripotent properties of these cells and development of approaches to direct their differentiation reproducibly into desired cell types at high efficiency. Most commonly, mouse embryonic fibroblast (MEF) feeder cells are employed to provide an environment that is suitable, although not necessarily optimal, for the maintenance of stem cell pluripotency. Routine MEF culture with medium containing animal-derived products carries the potential risk of animal pathogen or antigen transfer. To minimize such xeno-transfer, human feeder cells and autologous feeders created by differentiating hESCs have been developed (3–5). Nonetheless, the use of any feeder cell still retains the requirement for pathogen testing and does not avoid issues of undefined culture conditions and batch-to-batch variation. As an alternative approach, feeder-free cultures using different mixtures of defined medium and human or recombinant ECM components eliminate the risk of xenogeneic transfer and at the same time increase reproducibility (6–8). Ideally, an optimized culture system needs to be established that is xeno-free for applications such as future clinical therapies. The most successful early attempts at replacing feeders used Matrigel, an ill-defined basement membrane matrix derived from a mouse sarcoma cell line, generally together with feeder-conditioned medium (9–11). This system still retains the possibility of xenopathogen transfer and batch variation. However, newer defined serum-free media have now been developed that avoid the need for conditioning.

Our understanding of how hESCs are regulated *in vivo* is limited because of their transient nature and their tendency to differentiate easily (12). However, observations *in vitro* indicate that stem cell fate is controlled by many factors, both intrinsic genetic and epigenetic signals and extrinsic regulators, such as growth factors and extracellular matrix (ECM) components. Although much attention has been paid to the influence of growth factors on stem cell fate (6, 12), the role of the ECM has been relatively neglected. ECM components, which form dynamic adhesive structures that affect cell proliferation, survival, shape, migration, and differentiation, are important candidates for establishing an optimized feeder-free hESC culture system (13–16). In our laboratory, we developed a defined culture medium, which allows maintenance of several hESC lines for at least 15 passages (8). Using this system, we showed that hESCs grow well on human plasma fibronectin (8). Other studies have also reported the maintenance of stem cells using fibronectin or laminin substrates (6, 17), and more recently, these molecules have been used together for suspension culture of stem cells (18). In addition, other ECM molecules, such as vitronectin, have been shown to support stem cell self-renewal (8, 19, 20), and hESC culture on ECM derived from MEF feeders has been reported (21). Therefore, we set out to analyze comprehensively the ECM of hESC-supportive feeder cells using a proteomic approach.

Several previous studies have used proteomic approaches to identify proteins that regulate stem cell pluripotency. Some studies analyzed stem cell-conditioned Matrigel (22) or medium conditioned by feeder cells capable of maintaining hESCs (23, 24), whereas others analyzed membrane proteins of hESCs (25–27) or the hESC phosphoproteome (28, 29). Here, we used an MS-based proteomic approach to identify ECM proteins released by mouse and human feeders in order to characterize the range of ECM components that support the growth of self-renewing hESCs. We aimed to determine both similarities and differences between supportive and unsupportive feeder cells and so to dissect important and novel components of the ECM that maintain the pluripotent self-renewing state. We compared ECM derived from conventional MEFs, primary human placental stromal fibroblasts (hPSFs), and immortalized human placental stromal fibroblasts (ihPSFs) produced in our laboratory, which have been shown to support pluripotent hESC growth for over 25 passages (30). All tested mouse and human feeder cells supported hESC self-renewal, but only ECM derived from CD1×CD1 (referred to herein as CD1) MEFs or ihPSFs supported hESC self-renewal, whereas ECM derived from MF1×CD1 MEFs or hPSFs was unsupportive. We found that many ECM proteins are expressed by both mouse and human feeders and are also produced by hESCs. Intriguingly, quantitative differences were identified between supportive and unsupportive matrices, and some proteins were only detected in supportive ECMs; these proteins might play a role in the maintenance of pluripotency. We tested candidate ECM molecules, including perlecan, fibrillin-1, fibulin-2, collagen VI, and tenascin C, as substrates for feeder-free growth of hESCs. Our results show that some of these molecules can support attachment and self-renewal of hESCs alone or in combination with a low, unsupportive concentration of fibronectin, in the absence of feeders. Thus, this study further illuminates the role that ECM interactions play in the hESC phenotype, which has until recently been a neglected area of hESC biology.

## EXPERIMENTAL PROCEDURES

### 

#### 

##### Fibroblast Cell Culture

MEFs were prepared from 13.5-day embryos from CD1×CD1 (referred to herein as CD1) or MF1×CD1 mice (8, 31). For preparation of feeders used for hESCs, MEFs were cultured to passage 4 (P4), P9, or P14 and were mitotically inactivated using 10 μg/ml mitomycin C (Sigma-Aldrich). hPSFs and ihPSFs were cultured to P10 and P10(6), respectively, according to Camarasa *et al.* (32) and McKay *et al.* (30), respectively, in DMEM (PAA Laboratories) supplemented with 10% (v/v) FCS (Invitrogen), 2 mm l-glutamine (PAA Laboratories), and 0.5% (v/v) penicillin/streptomycin (PAA Laboratories).

##### hESC Culture on Feeders

hESC lines HUES1 and HUES7 (33) were cultured on inactivated MEFs derived from CD1 or MF1×CD1 mice. Twenty-four hours before hESC culture, MEFs at P4, P9, or P14 were plated (2–3 × 10^4^ cells/cm^2^) onto tissue culture plates coated with 0.1% (w/v) gelatin (Sigma-Aldrich). hESCs were cultured on MEFs in knock-out DMEM (PAA Laboratories) supplemented with 20% (v/v) knock-out serum replacement, 100 μm β-mercaptoethanol (Invitrogen), 1× non-essential amino acids (PAA Laboratories), 2 mm l-glutamine, 0.5% (v/v) penicillin/streptomycin, and 10 ng/ml bovine fibroblast growth factor (FGF) (Autogen Bioclear). Trypsin/EDTA (PAA Laboratories) was used to passage hESCs, as described previously (34). hESCs were cultured on hPSFs and ihPSFs as on MEFs, except that hESCs were first cultured on inactivated MEF feeders and then transferred onto inactivated PSFs after they reached confluence. hESCs were cultured for 5 days before passage.

##### hESC Culture without Feeders

HUES1 cells were cultured on tissue culture plates coated with 50 μg/ml human plasma fibronectin (Millipore) in 50:50 F-12/DMEM supplemented with 0.1% (w/v) BSA (Sigma-Aldrich), 100 μm β-mercaptoethanol, 1× non-essential amino acids, 2 mm l-glutamine, 20 ng/ml bovine FGF, 1× N2 supplement (Invitrogen), 1× B27 supplement (Invitrogen), 100 ng/ml activin A (R&D Systems), and 2 ng/ml NT4 (Peprotech) (modified from Ref. 8). When cells were ∼90% confluent, they were passaged in a 1:3 ratio using TrypLE Express (Invitrogen).

##### hESC Culture on Cell-derived ECM

MEFs at P4, P9, or P14, hPSFs, or ihPSFs were plated (6.8 × 10^4^ cells/cm^2^) onto tissue culture plates coated with 0.1% (w/v) gelatin. Fibroblast cells were cultured for 14 days, and then ECM was denuded by incubating cells with extraction buffer (20 mm NH_4_OH, 0.5% (w/v) Triton X-100 (Sigma-Aldrich) in PBS) for 2 min at room temperature or until no intact cells were visible. Plates were washed three times with PBS and used immediately for culturing hESCs or stored at 4 °C in 1% (v/v) penicillin/streptomycin. The hESC line HUES1 was cultured on ECM derived from MEFs at P4, primary hPSFs at P10, or immortalized ihPSFs at P10(6) in feeder-free medium (as described above) and passaged using TrypLE Express.

##### hESC Culture on Defined Substrates

Twenty-four-well tissue culture plates were incubated with 100 μg/ml collagen VI (BD Biosciences), 50 μg/ml tenascin C (Millipore), 10 or 20 μg/ml fibulin-2 (courtesy of T. Sasaki, Max Planck Institute of Biochemistry), 10 or 20 μg/ml fibrillin-1 (recombinant fragment PF17; courtesy of S. A. Cain and C. M. Kielty, University of Manchester) (35), 20 μg/ml perlecan (courtesy of J. M. Whitelock, University of New South Wales), 10 μg/ml versican (Novus Biologicals), or 10 μg/ml biglycan (Sigma-Aldrich) overnight at 4 °C. For substrates tested in combination with fibronectin, plates were then incubated with 5 μg/ml human plasma fibronectin overnight at 4 °C. Feeder-free HUES1 or HUES7 cells were dissociated using TrypLE Express and plated (1 × 10^6^ cells/cm^2^) onto 24-well tissue culture plates in feeder-free medium. Cells were cultured on each substrate for three consecutive passages before assessing their expression of pluripotency-associated genes by immunocytochemistry. Cells cultured on 5 and 50 μg/ml fibronectin were used as controls in each experiment.

##### Immunocytochemistry

Cells plated on fibronectin-coated wells or glass coverslips were fixed with 4% (w/v) paraformaldehyde for 10 min at room temperature, blocked, and stained. Primary antibodies and concentrations used were anti-Nanog (2 μg/ml; R&D Systems); anti-Oct4 (2.5 μg/ml; BD Biosciences); anti-TRA-1-81 (1 μg/ml; Abcam); anti-Sox2, anti-GATA4, anti-α-smooth muscle actin, anti-βIII-tubulin, anti-Sox17, anti-brachyury, anti-vimentin, and anti-α-fetoprotein (all 5 μg/ml; all R&D Systems); anti-fibronectin (1.25 μg/ml; Sigma-Aldrich); anti-tenascin C (10 μg/ml; Millipore); anti-collagen VI (10 μg/ml; Abcam); anti-human collagen XII and anti-mouse collagen XII (1:3000 and 1:1000, respectively; kind gift of M. Koch, University of Cologne); pan-specific anti-laminin (1:100; kind gift of D. R. Garrod, University of Manchester); anti-fibrillin-1 N-19 (N-terminal region) and PRO (proline-rich region) (1:50 and 1:200, respectively; kind gift of C. M. Kielty); and anti-fibulin-2 (2.5 μg/ml; kind gift of T. Sasaki). Secondary antibodies used were Alexa Fluor 488- and Alexa Fluor 594-conjugated anti-Ig (Invitrogen). Cells were mounted in Vectashield hardset mounting medium with DAPI (Vector) and examined using a fluorescence microscope (Olympus) equipped with ×10, ×20, and ×40 objective lenses and a digital camera (QImaging).

##### Flow Cytometry

Cells were dissociated using TrypLE Express, washed with PBS, and resuspended to 1 × 10^7^ cells/ml in PBS containing 1% (v/v) FCS (PBS-FCS). Primary antibodies (10 μg/ml, final concentration) in PBS containing 0.02% (w/v) sodium azide were added to cells, and samples were incubated overnight at 4 °C and then washed three times with PBS-FCS. Cells were resuspended in FITC- or Alexa Fluor-conjugated secondary antibody in PBS-FCS and incubated for 45 min at 4 °C. Samples were washed three times with PBS-FCS and then fixed with 0.4% (w/v) formaldehyde in PBS. IgG and secondary antibodies alone were used as controls. Samples were analyzed using a CyAn ADP flow cytometer (Beckman Coulter).

##### Isolation of Cell-derived ECM

Cell-derived ECM was derived as described above, treated with 10 units/ml DNase I (Promega) for 30 min at 37 °C, and then washed two times with PBS. ECM was solubilized with prewarmed 2× reducing sample buffer (50 mm Tris-HCl, pH 6.8, 10% (w/v) glycerol, 4% (w/v) SDS, 0.004% (w/v) bromphenol blue, 8% (v/v) β-mercaptoethanol) and removed from the plate using a cell scraper. Proteins were resolved by SDS-PAGE and stained with Coomassie Brilliant Blue. ECMs extracted from equal numbers of cells were compared.

##### Embryoid Body Formation

HUES1 or HUES7 cells were cultured for at least five passages on ECM derived from CD1 P4 MEFs or ihPSFs, dissociated using TrypLE Express, and cultured for 10 days in DMEM supplemented with 20% (v/v) FCS in bacteriological grade culture dishes. Day 10 embryoid bodies were plated on 12-well plates coated with 0.1% (w/v) gelatin in DMEM supplemented with 20% (v/v) FCS and cultured for a further 14 days.

##### Karyotypic Analysis

HUES1 or HUES7 cells were cultured for three passages in feeder-free conditions. hESCs were prepared for karyotyping as described previously (8) and examined for karyotypic abnormalities by The Doctors Laboratory Genetics.

##### MS Data Acquisition and Analysis

Solubilized ECM proteins were subjected to in-gel tryptic digestion as described by Shevchenko *et al.* (36) with modifications to enable processing in 96-well plates as described by Humphries *et al.* (37). Analysis of peptides by LC-MS/MS was performed using a nanoACQUITY UltraPerformance LC system (Waters) coupled online to a 4000 Q TRAP triple-quadrupole linear ion trap analyzer (Applied Biosystems), as described previously (37). Peak list files were searched against a modified version of the IPI Human database (version 3.70, release date March 4, 2010) containing 10 additional contaminant and reagent sequences of non-human origin or the IPI Mouse database (version 3.70, release date March 4, 2010). Searches were submitted to an in-house Mascot server (version 2.2.03; Matrix Science) (38). Carbamidomethylation of cysteine was set as a fixed modification; oxidation of methionine and hydroxylation of proline and lysine were allowed as variable modifications. Only tryptic peptides were considered, with up to one missed cleavage permitted. Monoisotopic precursor mass values were used, and only doubly and triply charged precursor ions were considered. Mass tolerances for precursor and fragment ions were 1.5 and 0.5 Da, respectively. Rigorous statistical algorithms at both the peptide and protein level were employed to validate the proteomic data sets generated by MS (39, 40). To achieve this, data validation was performed using Scaffold (version Scaffold_3_00_06; Proteome Software), as described by Humphries *et al.* (37). Protein identifications were accepted if they were assigned at least two unique validated peptides (established with at least 90% probability) and had a protein probability of at least 99%. These acceptance criteria resulted in an estimated protein false discovery rate of 0.1% for all data sets. MS data were converted using PRIDE Converter (version 2.5.4) (41) and deposited in the PRIDE database (42) under accession numbers 19910–19937. Details of all identified proteins and peptides are provided in supplemental Files S1-S3.

Quantification of relative protein abundance was performed using spectrum counting (43–45). Relative protein abundance was calculated on the basis of the unweighted spectrum count assigned to each identified protein by Scaffold. To normalize the data, spectrum counts were expressed as a percentage of the total number of spectra observed in the entire sample. Mean normalized spectrum counts were calculated using data from two independent ECM isolations.

##### Bioinformatic Data Analysis

Hierarchical clustering analysis was performed as described previously (37, 46). Gene Ontology enrichment analysis was performed using DAVID Bioinformatic Resources (version 6.7) (47). Gene Ontology annotations of proteins were assembled from the UniProt Knowledgebase Gene Ontology Annotation database (48) accessed using QuickGO (49). Proteins annotated with Gene Ontology terms GO:0005576 (extracellular region), GO:0005615 (extracellular space), GO:0005886 (plasma membrane), or GO:0009986 (cell surface) were classified as extracellular or cell surface and visualized as protein-protein interaction networks. Interaction network analysis was performed using Cytoscape (version 2.8.1) (50). Protein hits were mapped onto a merged human interactome built from the Protein Interaction Network Analysis platform *Homo sapiens* network (release date March 4, 2010) (51), the ECM interactions database MatrixDB (release date August 26, 2010) (52), and a literature-curated database of integrin-based adhesion-associated proteins (53). Proteins in the mouse data set were converted to human orthologs using InParanoid (54). Interaction networks were clustered using the yFiles Organic algorithm implemented in Cytoscape, and topological parameters were computed using the NetworkAnalyzer plug-in (55). Interaction network models are provided in supplemental Figs. S4 –S8.

## RESULTS

### 

#### 

##### Culture of hESCs on Mouse and Human Feeders

hESCs are routinely cultured on mitotically inactivated MEF feeders, and it has been suggested that MEFs beyond P4 or P5 are unable to support pluripotency of hESCs (56). To assess the ability of CD1 and MF1×CD1 MEF strains used in this study to support stem cell maintenance, HUES1 cells were cultured on MEFs that had been inactivated at either P4 or P9. After culture for three passages, all HUES1 colonies displayed nuclear immunostaining of the pluripotency-associated markers Oct4 and Nanog, indicating stem cell maintenance on both MEF strains at both passages (supplemental Fig. S1). To assess the ability of hPSFs and ihPSFs to support maintenance, HUES1 cells were cultured on hPSFs or ihPSFs for three passages. HUES1 colonies grown on either hPSFs or ihPSFs displayed nuclear expression of Oct4 and Nanog, indicating that stem cells were maintained on these human feeders (supplemental Fig. S1).

##### Culture of hESCs on Cell-derived ECM

**FIGURE 1.**
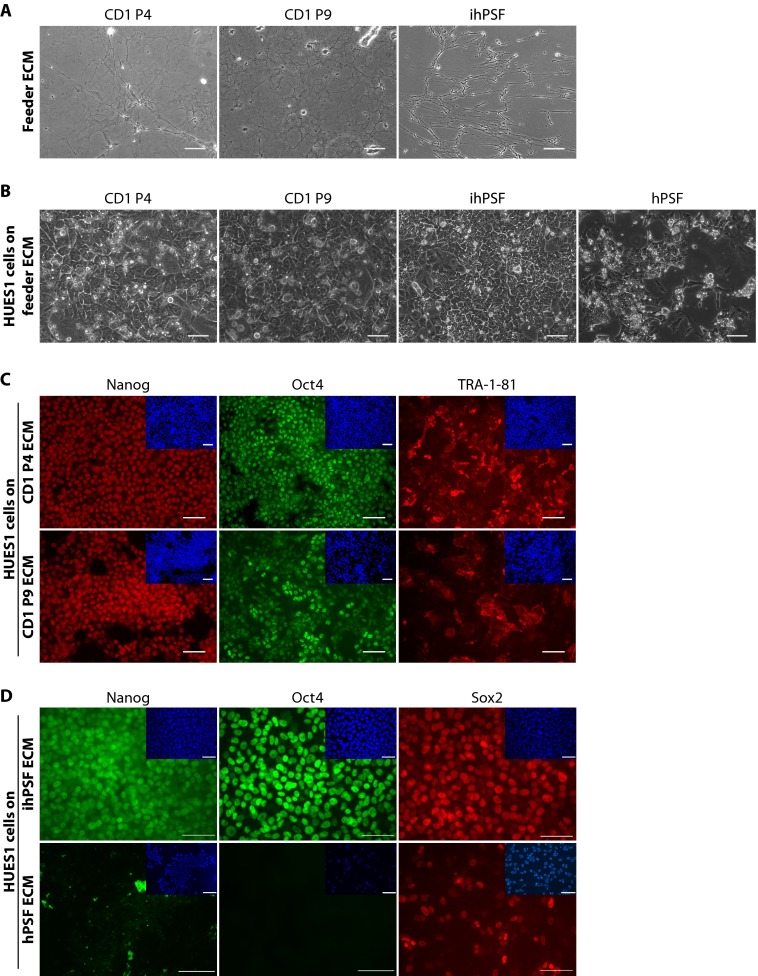
**Culture of hESCs on feeder cell-derived ECM.** *A*, phase-contrast images of ECMs isolated from CD1 MEFs at P4 and P9 and from ihPSFs by alkali/detergent extraction. *Scale bars*, 100 μm. *B*, phase-contrast images showing morphology of HUES1 cells cultured for five consecutive passages on ECM derived from CD1 P4 and P9, ihPSF, and hPSF. *Scale bars*, 100 μm. *C*, HUES1 cells cultured on CD1 P4 and P9 ECM stained positively for pluripotency-associated markers Nanog and Oct4 and surface marker TRA-1-81 after multiple (up to four) passages. Cell nuclei were stained with DAPI (*blue*; *insets*). *Scale bars*, 100 μm. *D*, HUES1 cells cultured on ihPSF-derived ECM stained positively for pluripotency-associated markers Nanog, Oct4, and Sox2 after multiple (up to four) passages, whereas HUES1 cells cultured on hPSF-derived ECM lost the expression of pluripotency-associated markers Nanog, Oct4, and Sox2. Cell nuclei were stained with DAPI (*blue*; *insets*). *Scale bars*, 50 μm.

In order to assess the role of ECM macromolecules in the support of stem cell maintenance, HUES1 cells were cultured for at least four passages in feeder-free medium on ECM isolated from mouse or human feeder monolayers by alkali/detergent extraction. After extraction, the remaining deposited ECM was observable by phase-contrast microscopy at both P4 and P9 (Fig. 1*A*). ECM derived from CD1 MEFs at either P4 or P9 supported HUES1 cell attachment and culture expansion. HUES1 cells cultured on CD1 ECM for five consecutive passages formed monolayers and exhibited typical morphological features of hESCs cultured on MEFs, with a high nucleus-to-cytoplasm ratio and prominent nucleoli (Fig. 1*B*). Cells were also positive for the pluripotency-associated markers Nanog and Oct4 and the surface marker TRA-1-81 (Fig. 1*C*). Neither P4 nor P9 MF1×CD1 ECM supported self-renewal of HUES1 cells for four passages (five independent experiments). ECMs derived from CD1 and MF1×CD1 MEFs at P14 were also assessed but were not supportive for hESC maintenance (data not shown).

HUES1 cells attached to the ECM deposited by ihPSFs (P10(6)) and, to a lesser extent, to the ECM from hPSFs (P10) (Fig. 1*B*). hESCs grown for three passages on ihPSF ECM displayed typical hESC morphology, with a high nucleus/cytoplasm ratio and prominent nucleoli as well as nuclear expression of Nanog, Oct4, and Sox2 (Fig. 1, *B* and *D*). However, ECM derived from hPSF (P10) failed to maintain the self-renewal of HUES1 cells for three passages, as indicated by a change in morphology from rounded to a more spread shape with cell extension and by the loss of Nanog, Oct4, and Sox2 nuclear expression (Fig. 1, *B* and *D*). Together, these findings indicated that the ECMs produced by the primary and immortalized human feeders were qualitatively or quantitatively different with respect to components that support hESC maintenance.

To confirm the *in vitro* differentiation potential of hESCs into multiple cell lineages, embryoid bodies were generated from hESCs that had been cultured on ECM derived from either CD1 MEFs or ihPSFs. Immunostaining indicated the ability of these hESCs to give rise to derivatives of all three germ layers (supplemental Fig. S2).

##### Integrin Expression in hESCs

Because cells interact with the ECM predominantly via integrin receptors, we used flow cytometry to establish the expression of a number of integrins by hESCs cultured feeder-free on fibronectin. Integrin αvβ3 was not detected, but integrin chains α1, α2, α5, α6, and β1 and integrin αvβ5 were all expressed in HUES1 cells (supplemental Fig. S3). These data show that, in addition to the fibronectin receptor integrin α5β1 (which also interacts with other ligands, such as fibrillin-1 and osteopontin) (57), hESCs express receptors for several ECM ligands, including collagen (integrins α1β1 and α2β1) and laminin (integrins α1β1, α2β1, and α6β1).

##### Proteomic Analysis of Cell-derived ECM

**FIGURE 2.**
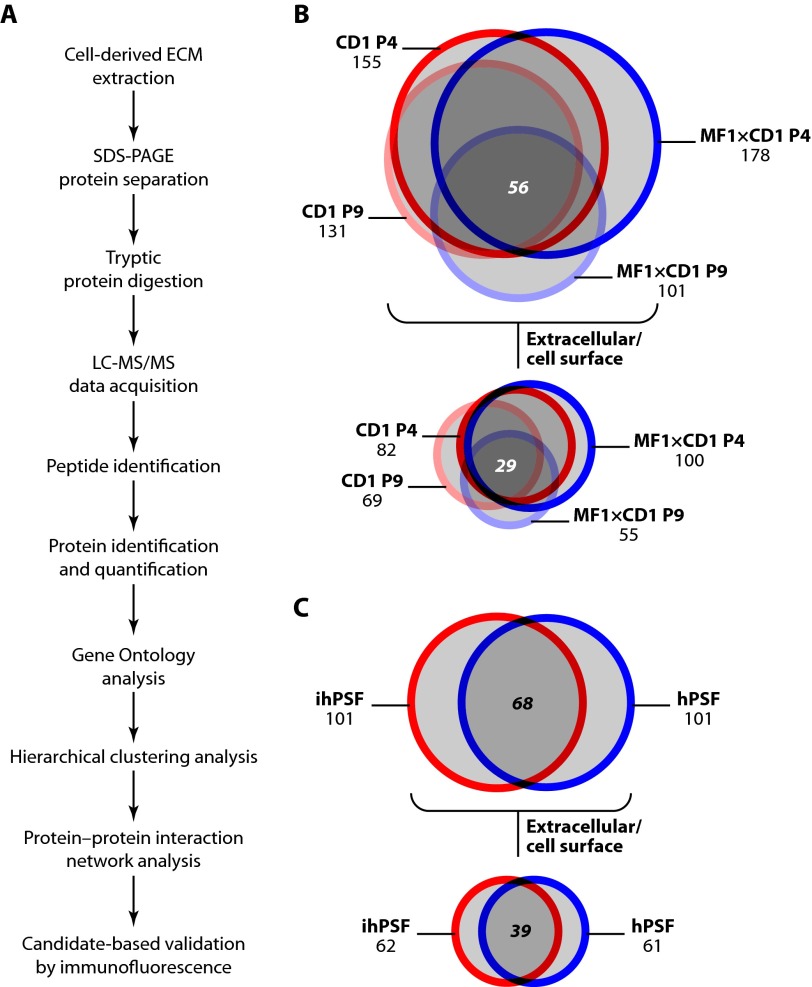
**Proteomic analysis of feeder cell-derived ECM.** ECM isolated from feeder cells by alkali/detergent extraction was analyzed by MS and bioinformatics. *A*, the workflow of analysis of ECM by MS-based proteomics. *B* and *C*, area-proportional Euler diagrams display the overlap of proteins identified by proteomic analysis of ECM isolated from equal numbers of mouse (*B*) and human (*C*) feeder cells. Proteins classified as extracellular or cell surface (as described under “Experimental Procedures”) are represented in associated Euler diagrams. Sets are labeled with feeder cell types and cardinalities; *boldface italic* type indicates intersection cardinalities, which represent the numbers of proteins identified in multiple ECMs.

We used MS-based proteomics to catalogue the components of ECM isolated from mouse and human feeder cells (Fig. 2*A*). MS analysis identified 155 and 131 proteins in ECM derived from CD1 MEFs at P4 and P9, respectively, and 178 and 101 proteins in ECM derived from MF1×CD1 MEFs at P4 and P9, respectively (*n* = 2; Fig. 2*B*). Of these proteins, 82 and 69 were classified by Gene Ontology analysis as extracellular/cell surface proteins in ECM derived from CD1 MEFs at P4 and P9, respectively, and 100 and 55 were classified as extracellular/cell surface proteins in ECM derived from MF1×CD1 MEFs at P4 and P9, respectively (Fig. 2*B*). MS analysis identified 101 proteins in ECM derived from each of hPSFs and ihPSFs (*n* = 2; Fig. 2*C*). Of these proteins, 61 and 62 were classified as extracellular/cell surface proteins in ECM derived from hPSFs and ihPSFs, respectively (Fig. 2*C*). The numbers of identified proteins were comparable with other MS-based proteomic analyses of ECMs (58). Indeed, of the total protein identifications in our data sets, 34 ± 5.3% (mean ± S.D.) were classified as extracellular proteins (56 ± 3.8% were classified as extracellular or cell surface proteins), which compares favorably with the 12–30% enrichment of ECM proteins reported in previous proteomic studies (58). Furthermore, there was substantial overlap between extracellular/cell surface proteins identified in different feeder cell ECMs, as shown by the intersection sets of the Euler diagrams (Fig. 2, *B* and *C*). This suggests that, in addition to some qualitative differences in the ECMs, differences in the amounts of proteins incorporated into distinct ECMs may contribute to hESC self-renewal.

**FIGURE 3.**
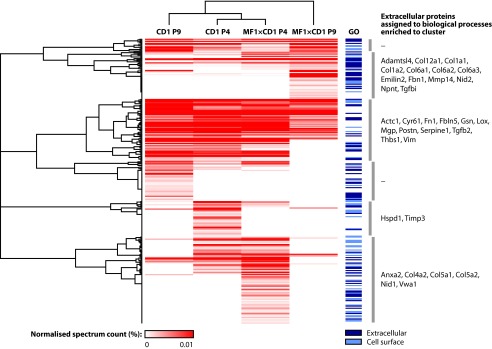
**Hierarchical clustering of proteins identified by proteomic analysis of mouse feeder cell-derived ECM.** Unsupervised hierarchical clustering analysis was used to group proteins identified by MS analysis. The quantitative heat map displays mean spectrum counts normalized to the total number of spectra identified in each analysis. Associated dendrograms display hierarchical clustering on the basis of uncentered Pearson correlation. *Blue bars*, proteins classified as extracellular (*dark blue*) or cell surface (*light blue*). *Gray vertical bars*, clusters based on similar enrichment profiles across the different samples and a Pearson correlation of at least 0.5. Proteins in each cluster were subjected to functional enrichment analysis using the Gene Ontology biological process domain. Extracellular proteins assigned to significantly enriched Gene Ontology terms (Benjamini-Hochberg-corrected *p* ≤ 0.05) are listed as gene names *beside* their respective clusters. Enriched Gene Ontology terms are detailed in supplemental Table S3.

**FIGURE 4.**
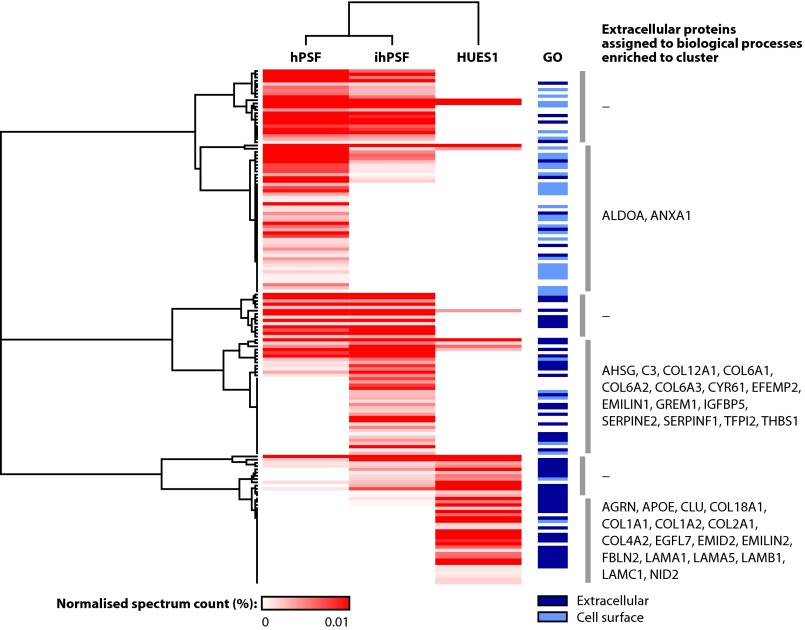
**Hierarchical clustering of proteins identified by proteomic analysis of human feeder cell- and hESC-derived ECM.** Unsupervised hierarchical clustering analysis was used to group proteins identified by MS analysis. The quantitative heat map displays mean spectrum counts normalized to the total number of spectra identified in each analysis. Associated dendrograms display hierarchical clustering on the basis of uncentered Pearson correlation. *Blue bars* indicate proteins classified as extracellular (*dark blue*) or cell surface (*light blue*). *Gray vertical bars* indicate clusters based on similar enrichment profiles across the different samples and a Pearson correlation of at least 0.5. Proteins in each cluster were subjected to functional enrichment analysis using the Gene Ontology biological process domain. Extracellular proteins assigned to significantly enriched Gene Ontology terms (Benjamini-Hochberg-corrected *p* ≤ 0.05) are listed as gene names *beside* their respective clusters. Enriched Gene Ontology terms are detailed in supplemental Table S4.

To aid the interrogation and visualization of the MS data sets and to highlight quantitative differences between them, hierarchical clustering was performed to detect patterns in the data. Unsupervised clustering identified clusters of proteins enriched in different cell-derived ECMs (Figs. 3 and 4 and supplemental Tables S1 and S2). For ECM isolated from mouse feeders, large contiguous clusters of proteins were shared between ECMs from different MEF populations (Fig. 3). As for many of the clusters, there was an overrepresentation of proteins involved in processes such as cell adhesion and ECM organization (*e.g.* fibronectin, periostin, and thrombospondin-1) in the main cluster of shared proteins (Fig. 3, *third from top* cluster), as determined by Gene Ontology enrichment analysis (supplemental Table S3). In addition, several clusters of proteins were enriched in distinct samples, suggesting that the relative abundance of these proteins may play a role in the support of hESC maintenance. For example, clusters of proteins enriched in MF1×CD1 ECM comprised additional collagens (Fig. 3, *second from top* cluster and *bottom* cluster, and supplemental Table S3). For ECM isolated from human feeders, there were shared and unique clusters of proteins (Fig. 4), as for mouse feeder ECM. Interestingly, protein clusters enriched in ECM derived from ihPSF contained more proteins annotated as extracellular, whereas clusters enriched in hPSF ECM contained more proteins annotated as cell surface (Fig. 4 and supplemental Table S2). Furthermore, there was an overrepresentation of proteins involved in cell adhesion and ECM organization in the major ihPSF-enriched cluster (Fig. 4, *fourth from top* cluster, and supplemental Table S4) but not in the major hPSF-enriched cluster (Fig. 4, *second from top* cluster, and supplemental Table S4). These data suggest that ihPSFs, which support hESC maintenance, secrete more structural ECM components to produce a more complex fibrillar network compared with hPSFs, which do not support hESC self-renewal (30).

In order to assess whether hESCs secrete ECM proteins that may facilitate the maintenance of pluripotency, we analyzed ECM derived from HUES1 cells cultured on fibronectin-coated plates for five or seven passages by MS, conditions that we have previously shown to maintain self-renewing hESCs (8). A total of 77 proteins were identified in hESC ECM (*n* = 2), of which 35 were classified as extracellular/cell surface proteins (Fig. 4 and supplemental Table S2). Moreover, proteins involved in cell adhesion and ECM organization were overrepresented in the major HUES1-enriched cluster (Fig. 4, *bottom* cluster, and supplemental Table S4). Many of the proteins present in the ECM isolated from mouse and human feeders were also present in ECM derived from hESCs (Table 1). HUES1 ECM shared a similar number of proteins with CD1 and MF1×CD1 MEFs at P4 and P9 (13 ± 1.6 extracellular proteins; 37% ± 4.7%; mean ± S.D.). HUES1 ECM shared more proteins with ihPSF ECM (20 extracellular proteins; 57%) than it did with hPSF ECM (10 extracellular proteins; 29%). Notably, laminin chains, fibrillin-1, nidogen-1, collagen IV, and perlecan were detected in HUES1 and ihPSF ECMs but not in hPSF ECM (Table 1). In addition, collagen XII was detected in ihPSF ECM but not in hPSF ECM. Furthermore, collagen I, collagen VI, CYR61, EMILIN-1, fibronectin, and thrombospondin-1 were enriched in ihPSF ECM (greater than 2-fold) compared with hPSF ECM. Fibronectin was enriched in ihPSF ECM 24-fold over hPSF ECM (Table 1). These qualitative and quantitative differences in the composition of ECM from supportive and non-supportive feeder cells may play a role in the maintenance of stem cell pluripotency.

**TABLE 1:** **Extracellular proteins detected in HUES1 ECM compared with feeder ECMs** Cell-derived ECM was isolated and analyzed by MS as described under “Experimental Procedures.” Proteins assigned to the Gene Ontology terms GO:0005576 (extracellular region), GO:0005615 (extracellular space), GO:0005886 (plasma membrane) or GO:0009986 (cell surface) are listed. Additional collagen chains not detected in HUES1 ECM (italic type) are listed for reference. Complete data sets are provided as supplemental Tables S1 and S2. ADAM-TS, a disintegrin and metalloproteinase (ADAM) metallopeptidase with thrombospondin type I motif; hnRNP, heterogeneous nuclear ribonucleoprotein; ND, not detected.

Gene symbol	Protein name	Normalized spectrum count						
		CD1 P4	CD1 P9	MF1×CD1 P4	MF1×CD1 P9	hPSF	ihPSF	HUES1
		% *total spectra* × *10^3^*						
ADAMTS4	ADAM-TS4	ND	0.500	ND	ND	1.18	2.03	4.76
AGRN	Agrin	ND	ND	ND	ND	ND	ND	10.0
APOE	Apolipoprotein E	ND	ND	ND	ND	ND	ND	215
CLU	Clusterin	ND	ND	ND	ND	ND	ND	43.1
*COL12A1*	*Collagen, type XII,* α*1*	*2.60*	*1.91*	*8.23*	*6.44*	*ND*	*0.470*	*ND*
COL18A1	Collagen, type XVIII, α1	ND	ND	ND	ND	ND	ND	5.83
COL1A1	Collagen, type I, α1	ND	1.14	1.45	3.70	1.02	7.36	54.3
COL1A2	Collagen, type I, α2	0.439	0.520	1.34	3.26	ND	3.06	18.1
COL2A1	Collagen, type II, α1	ND	ND	ND	ND	ND	ND	1.2
*COL3A1*	*Collagen, type III,* α*1*	*ND*	*0.490*	*ND*	*ND*	*ND*	*ND*	*ND*
COL4A1	Collagen, type IV, α1	ND	ND	0.253	ND	ND	1.63	5.52
COL4A2	Collagen, type IV, α2	ND	ND	0.184	ND	ND	1.04	18.5
*COL5A1*	*Collagen, type V,* α*1*	*ND*	*ND*	*0.330*	*ND*	*ND*	*ND*	*ND*
*COL5A2*	*Collagen, type V,* α*2*	*ND*	*ND*	*0.640*	*ND*	*ND*	*ND*	*ND*
*COL6A1*	*Collagen, type VI,* α*1*	*ND*	*ND*	*0.800*	*1.46*	*0.740*	*5.23*	*ND*
*COL6A2*	*Collagen, type VI,* α*2*	*ND*	*ND*	*ND*	*0.960*	*0.980*	*4.49*	*ND*
*COL6A3*	*Collagen, type VI,* α*3*	*ND*	*ND*	*ND*	*ND*	*1.25*	*8.41*	*ND*
CYR61	CYR61	1.58	2.68	0.732	ND	21.5	47.0	2.55
EGFL7	EGF-like protein 7	ND	ND	ND	ND	ND	ND	12.5
EMID2	Collagen, type XXVI, α1	ND	ND	ND	ND	ND	ND	8.34
EMILIN1	EMILIN-1	13.7	27.3	13.0	34.7	24.9	49.7	13.2
EMILIN2	EMILIN-2	ND	ND	ND	1.25	ND	ND	1.39
FBLN2	Fibulin-2	54.4	31.8	32.8	69.6	ND	ND	6.13
FBN1	Fibrillin-1	0.274	ND	0.262	10.1	ND	0.375	3.09
FBN2	Fibrillin-2	ND	ND	ND	0.671	ND	ND	11.0
FGFBP3	FGF-binding protein 3	ND	ND	ND	ND	ND	ND	5.74
FN1	Fibronectin-1	99.1	117	95.1	118	2.34	57.2	81.9
HNRNPM	hnRNP M	ND	1.00	ND	ND	ND	ND	15.9
HSP90B1	Endoplasmin	ND	ND	ND	ND	1.00	1.19	4.08
HSPG2	Perlecan	6.63	13.1	10.3	15.8	ND	4.16	15.7
KRT1	Keratin, type II, cytoskeletal 1	8.78	12.9	6.65	11.6	107	70.5	13.9
LAMA1	Laminin α1	ND	ND	ND	ND	ND	ND	1.97
LAMA5	Laminin α5	ND	ND	ND	ND	ND	0.219	1.82
LAMB1	Laminin β1	ND	ND	ND	ND	ND	0.295	5.29
LAMC1	Laminin γ1	ND	ND	ND	ND	ND	0.329	10.8
LEFTY2	TGF-β4	ND	ND	ND	ND	ND	ND	16.7
NID1	Nidogen-1	ND	ND	1.08	ND	ND	0.645	2.59
NID2	Nidogen-2	0.370	0.731	ND	15.5	ND	ND	1.07
PKM2	Pyruvate kinase isozyme M1	ND	ND	0.796	ND	ND	1.76	4.62
THBS1	Thrombospondin-1	13.9	29.0	20.8	7.84	1.79	4.93	1.25
TNC	Tenascin C	1.17	3.21	2.91	4.58	12.3	17.2	4.23
VCAN	Versican	ND	ND	ND	ND	0.937	1.71	3.44

##### Interaction Network Analysis

**FIGURE 5.**
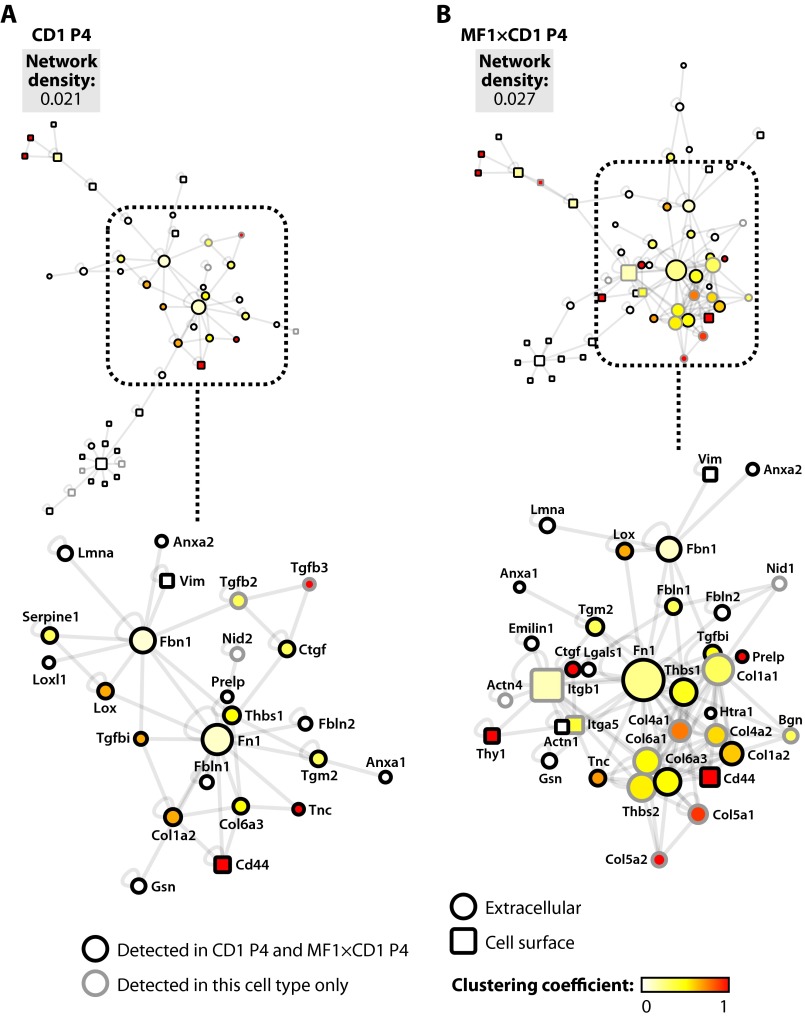
**Interaction network analysis of mouse feeder cell-derived ECM.** *A* and *B*, proteins classified as extracellular or cell surface were converted to protein-protein interaction network models. Interaction networks were clustered using the yFiles Organic algorithm. Topological parameters were computed for CD1 P4 (*A*) and MF1×CD1 P4 (*B*) ECM interaction networks as described under “Experimental Procedures.” Proteins identified by MS (nodes) are *colored* according to their clustering coefficient in each interaction network. Node diameter is proportional to the number of connected neighbors (degree). *Insets* show the most highly connected region of each interaction network in detail; for clarity, nodes are labeled with gene names. Disconnected nodes are not displayed. Proteins classified as extracellular are displayed as *circular nodes*; proteins classified as cell surface are displayed as *rectangular nodes*. Proteins detected in both CD1 P4 and MF1×CD1 P4 ECMs are indicated by a *black node border*; a *gray node border* indicates unique identification in ECM derived from that cell type.

The structure of biological networks derived from multiple protein-protein interactions has been shown to correlate with the functions of the constituent proteins (59, 60). To examine the molecular organization of the isolated ECM in the context of currently known protein-protein interactions, interaction network analysis was performed. To reduce the chance of selecting nonspecific cellular components for follow-up analysis, proteins classified as extracellular or cell surface were mapped onto a human interactome and topological parameters of the resultant interaction networks were computed. Interaction networks were visualized as graphs, with nodes representing proteins and edges representing protein-protein interactions. For ECM derived from mouse feeders, interaction networks for CD1 and MF1×CD1 MEFs had similar values for network density, a parameter that describes how densely a network is populated with protein-protein interactions (Fig. 5). Fibronectin and fibrillin-1 were major interconnected nodes in the interaction networks of both MEF strains, as indicated by their large number of interaction partners (large node size) and central “hub” positions, as determined by the network clustering algorithm. In support of the hierarchical clustering analysis (Fig. 3), the MF1×CD1 ECM interaction network contained additional collagen I and VI chains, collagens IV and V, and thrombospondin-2 (Fig. 5), which were not detected by MS in CD1 ECM. These additional molecules were highly clustered in the interaction network, as indicated by their large values for clustering coefficient, which is a measure of the interconnectedness of all of the interaction partners of a given protein. The additional molecules (with the exception of collagen V) had large numbers of interaction partners (large node sizes). Moreover, these molecules clustered around the fibronectin hub in the network and served to increase the number of interaction partners of molecules, such as fibronectin and thrombospondin-1, as compared with the CD1 ECM interaction network (Fig. 5). These findings suggest that the presence of additional collagens or thrombospondin-2 in MF1×CD1 ECM may change the network properties of the ECM, such that it is unable to support hESC maintenance, possibly by altering or inhibiting the interactions of a hub protein, such as fibronectin, with its binding partners.

**FIGURE 6.**
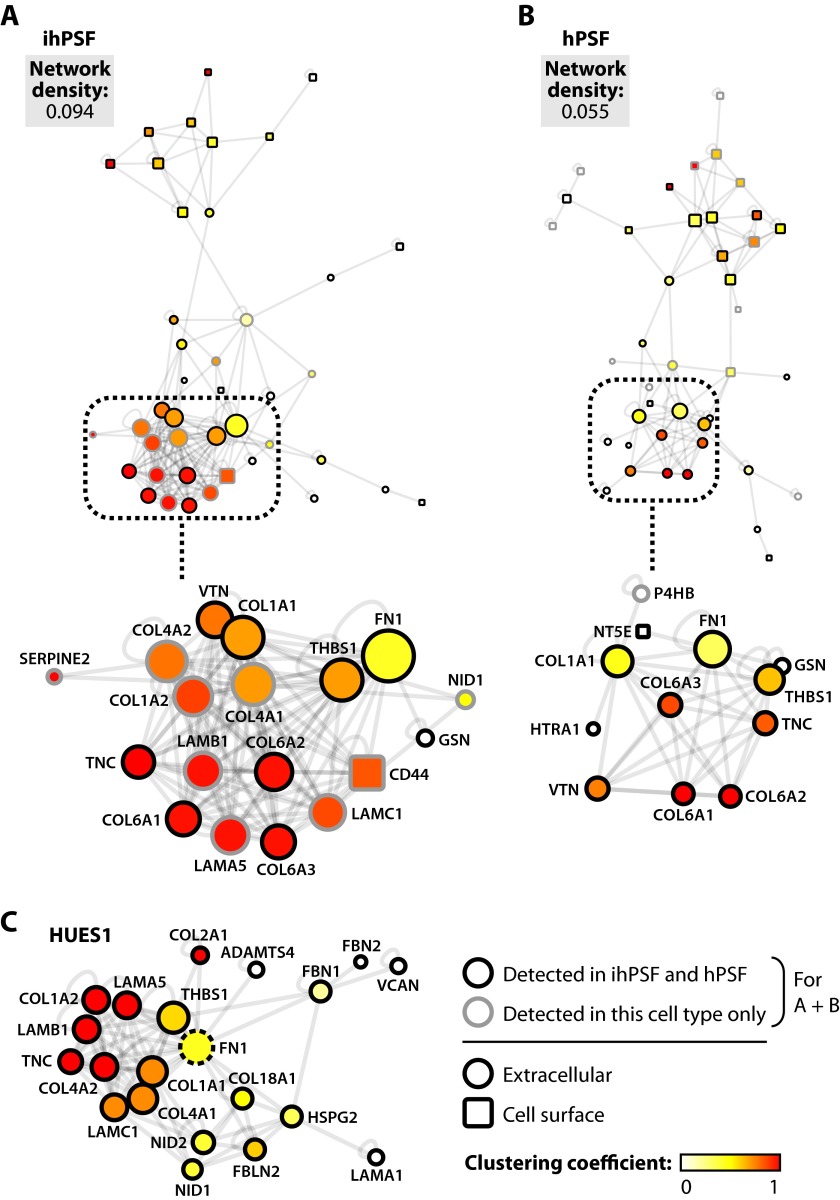
**Interaction network analysis of human feeder cell- and hESC-derived ECM.** *A–C*, proteins classified as extracellular or cell surface were converted to protein-protein interaction network models. Interaction networks were clustered using the yFiles Organic algorithm. Topological parameters were computed for ihPSF (*A*), hPSF (*B*), and HUES1 (*C*) ECM interaction networks as described under “Experimental Procedures.” Proteins identified by MS (nodes) are *colored* according to their clustering coefficient in each interaction network. Node diameter is proportional to number of connected neighbors (degree). *Insets* show the most highly connected region of each interaction network in detail (*A* and *B*); for clarity, nodes are labeled with gene names. Disconnected nodes are not displayed. Proteins classified as extracellular are displayed as *circular nodes*; proteins classified as cell surface are displayed as *rectangular nodes*. Proteins detected in both ihPSF and hPSF ECMs are indicated by a *black node border*; a *gray node border* indicates unique identification in ECM derived from that cell type (*A* and *B*). Fibronectin (*FN1*), which was used as a substrate to culture HUES1 cells in the absence of feeders, is indicated by a *dashed node border* (*C*).

For ECM derived from human feeders, the interaction network for ihPSFs had a notably denser, more interconnected network than that for hPSFs (Fig. 6, *A* and *B*). In the ihPSF interaction network, a highly clustered module around fibronectin contained additional collagen I chains, collagen IV, and laminins, which were not detected by MS in hPSF ECM. These proteins had high clustering coefficients and a large number of interaction partners, and they served to increase the number of interaction partners of neighboring molecules, as compared with the hPSF ECM interaction network. Although the presence of collagens was increased in the unsupportive MF1×CD1 mouse feeder ECM, the additional collagens in the human feeder ECM were detected in the supportive ihPSF ECM as compared with the unsupportive hPSF ECM. In addition, however, the ihPSF ECM contains laminins, which were not detected in the hPSF ECM and which have been shown previously to support stem cell self-renewal (61). Furthermore, interaction network analysis of ECM derived from HUES1 cells revealed a highly clustered network that had properties similar to those of the ihPSF interaction network (Fig. 6*C*). The HUES1 interaction network contained several collagens and laminins, which had large numbers of interaction partners. These findings suggest that ECM interactions that may be inhibitory to hESC growth, such as those potentially provided by collagens, may be overcome by the presence of key supportive components, such as laminin. Thus, the balance between ECM network properties and molecular composition appears critical for the support of hESC maintenance.

##### Validation of ECM Composition Using Immunocytochemistry

**FIGURE 7.**
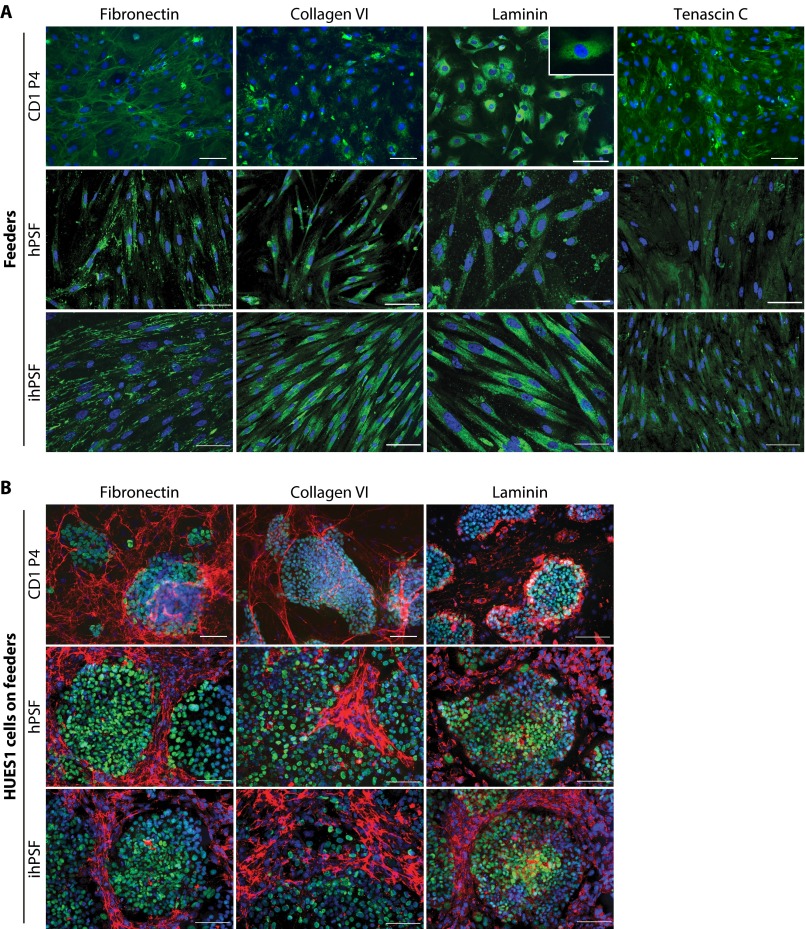
**ECM protein distribution in hESC culture on mouse and human feeder cells.** *A*, representative immunostaining of mouse and human feeder cells for fibronectin, collagen VI, laminin and tenascin C, which were among the major ECM components detected by MS analysis of either mouse or human feeder cell ECM in this study. Cell nuclei were stained with DAPI (*blue*). *Scale bars*, 50 μm. *B*, representative immunostaining images showing the distribution of fibronectin, collagen VI, and laminin associated with hESCs cultured on mouse and human feeder cells. ECM proteins (*red*) exhibited similar localization patterns around stem cell colonies when hESCs (positive for pluripotency-associated marker Oct4; *green*) were co-cultured with mouse and human feeders. Cell nuclei were stained with DAPI (*blue*). *Scale bars*, 100 μm.

In order to confirm the MS data, we performed immunostaining for candidate ECM molecules for which suitable antibodies could be obtained (Fig. 7). Both mouse and human feeders, cultured in the absence of hESCs, expressed fibronectin, collagen VI, and tenascin C (Fig. 7*A*), confirming data obtained using MS. Laminin staining was strong in human feeder ECMs but weaker and mostly cytoplasmic in mouse feeders, supporting the lack of its detection by MS in MEF ECM. Oct4-positive hESCs cultured in the presence of feeder cells appeared to assemble a fibrillar ECM network organization at the periphery of and surrounding the stem cell colony (Fig. 7*B*), whereas ECM secreted by feeder cells alone appeared to be more disorganized (Fig. 7*A*). No distinct organizational differences in ECM were observed between hESCs cultured on supportive or unsupportive feeder cells (Fig. 7*B*).

Expression of other ECM proteins was assessed in hESCs cultured on CD1 P4 MEFs (supplemental Fig. S4). Fibrillin-1 expression was cell-associated, restricted to hESC colonies, and colocalized with Oct4-positive cells. Extracellular fibulin-2 was seen as an extensive organized fibrillar network around Oct4-positive hESC colonies (supplemental Fig. S4). Immunostaining of Oct4-positive hESCs grown feeder-free on fibronectin for six passages revealed weak but detectable expression of tenascin C and collagen VI, confirming data obtained by MS (supplemental Fig. S5).

##### Feeder-free Culture of hESCs on Different Substrates

**FIGURE 8.**
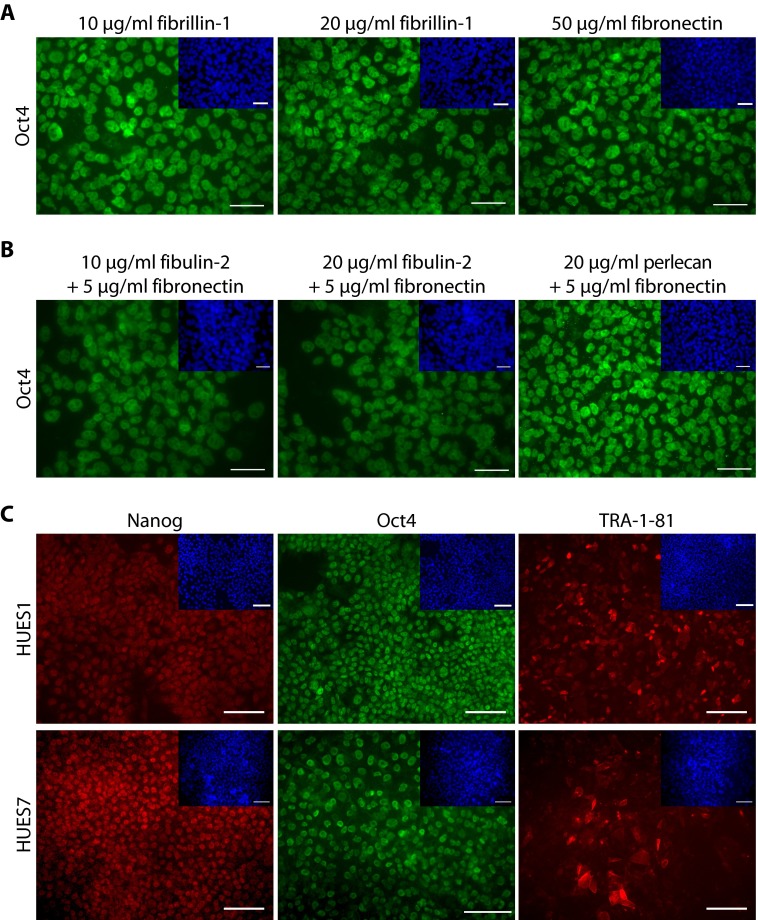
**Culture of hESCs on single ECM substrates or on substrates in combination with 5 μg/ml fibronectin.** *A*, HUES1 cells were successfully cultured over three passages on fibrillin-1 coated at two different concentrations (10 and 20 μg/ml), similar to feeder-free culture on fibronectin (50 μg/ml). *B*, fibulin-2 and perlecan supported HUES1 cell culture only in combination with 5 μg/ml fibronectin; on these substrates, hESCs were maintained over three passages. *C,* HUES1 and HUES7 cells were successfully cultured over five passages on fibrillin-1 (10 μg/ml) and were positive for pluripotency-associated markers Nanog and Oct4 and surface marker TRA-1-81. Cell nuclei were stained with DAPI (*blue*; *insets*). *Scale bars*, 100 μm.

We tested candidate molecules identified by MS analysis of supportive feeder or HUES1 cell ECMs as substrates for hESC growth. Supportive substrates were defined here as those that could maintain self-renewing hESCs expressing key pluripotency-associated markers for at least three passages in culture. Using feeder-free culture conditions (8), HUES7 exhibited a normal diploid karyotype, whereas that of HUES1 carried a single translocation, as present in the originally received hESCs (supplemental Fig. S6). Fibrillin-1, perlecan, fibulin-2, collagen VI, tenascin C, biglycan, and versican were tested as single substrates or in combination with a low, unsupportive concentration of fibronectin. Fibrillin-1 was identified as a major interconnected node in the interaction network analysis. As a single substrate, fibrillin-1 (at concentrations of 10 and 20 μg/ml) supported growth of pluripotent hESCs for three consecutive passages, as shown by nuclear expression of the pluripotency-associated marker Oct4 (Fig. 8*A*). Fibulin-2 and perlecan did not support hESC maintenance when used alone but were supportive for at least three passages in combination with 5 μg/ml fibronectin (Fig. 8*B*). Alone, 5 μg/ml fibronectin was unable to support growth of pluripotent hESCs (data not shown). When fibulin-2 and perlecan were tested alone, cells did not attach initially, but after 3 days, hESCs formed their own differentiated feeder-like cells, which allowed the remaining hESCs to attach and retain pluripotency-associated gene expression (data not shown). Subsequently, HUES1 and HUES7 were cultured for five passages on 10 μg/ml fibrillin-1. Similar to cells cultured on fibronectin, cells were passaged every 4–5 days, cell doubling time was ∼40 h, and cell viability was ∼75%. Cells were positive for pluripotency-associated markers Oct4 and Nanog and surface marker TRA-1-81 (Fig. 8*C*).

Collagen VI was tested as a substrate because of its potentially inhibitory role in hESC growth in MF1×CD1 MEF ECM and yet its abundant expression in supportive ihPSF ECM. When used as a single substrate, collagen VI did not support attachment of hESCs, which rather formed spherical suspended cell aggregates. Some cells in these aggregates retained expression of Oct4 for up to 15 days (Fig. 8*B*). hESCs plated on a combination of collagen VI and fibronectin attached more poorly than on fibronectin alone, without affecting pluripotency-associated marker expression (supplemental Fig. S7). hESCs did not adhere to tenascin C, versican, or biglycan and instead formed spherical cell clusters (data not shown). Other candidates, such as laminin-111 and -511, have been shown previously to support hESC self-renewal (61, 62) and so were not tested.

## DISCUSSION

The stem cell niche has been defined as a microenvironment that regulates stem cell self-renewal, proliferation, and differentiation via external signals, and its importance for proper stem cell function and fate determination is well established (61, 63, 64). hESCs are known to require precise conditions for culture and are routinely cultured in the presence of feeder cells, which provide a complex conditioning environment (34). However, there has been an increasing effort to refine hESC culture systems using defined conditions (with well established growth factors), including the use of single ECM substrates (6, 8, 19, 61). There is, therefore, a pressing need to analyze the ECM components produced by feeder cells that contribute to a favorable niche *in vitro* and to assess the contribution of individual ECM proteins to the support of stem cell maintenance. Here, we have employed a proteomic approach to identify the ECM components produced by feeder cells that maintain hESC self-renewal, by feeder cells that do not maintain self-renewal, and by hESCs cultured on a single, favorable substrate, fibronectin. We show that many ECM components are produced by supportive and unsupportive MEF and human PSF feeder cells, whereas some proteins are only expressed in supportive ECM, suggesting a role in the maintenance of hESC self-renewal. We demonstrate that, in the absence of feeders, fibrillin-1 alone and either perlecan or fibulin-2 in combination with fibronectin can support attachment and maintenance of hESCs. Together with interaction network analysis, these data highlight the importance of the balance between ECM network properties and molecular composition in the regulation of hESC phenotype and provide a resource for further studies of hESC self-renewal.

MS analysis of extracted ECMs revealed that both feeder cells and hESCs produce a complex network of ECM proteins. We showed that CD1 MEFs at P4 and P9 were supportive as feeders for hESC maintenance, as were the ECMs derived from these cells. MF1×CD1 MEFs at P4 and P9 were supportive as feeders, but their ECMs were unable to support hESCs in culture. Interaction network analysis revealed different network architectures between the ECMs of the two mouse feeder crosses. The MF1×CD1 ECM interaction network displayed a highly clustered module of collagens and thrombospondin-2, which was not present in the CD1 ECM network, which suggested that these molecules might play an inhibitory role in the support of hESC maintenance. This finding was supported by the loss of attachment of hESCs plated on collagen VI with fibronectin as compared with fibronectin alone. We speculate that this inhibition may arise by altering the interaction partners of a hub protein, such as fibronectin, which is known to play a key role in stem cell self-renewal (8, 65). Collagens and thrombospondins have been implicated in the maturation of cartilage by proteomic analysis of mouse neocartilage ECM (66). Furthermore, transforming growth factor β (TGF-β), a known regulator of hESC pluripotency (67), was enriched in CD1 ECM but absent from MF1×CD1 ECM, suggesting that insufficient levels of growth factors might also affect the supportive capacity of these ECMs.

ihPSFs have been previously shown to support hESC proliferation and self-renewal for up to 25 passages, whereas hPSFs could not (30). Here, we showed that ECM derived from ihPSFs was able to support hESC maintenance, whereas ECM from hPSFs was not. Our proteomic data revealed several differences between the composition of the ECMs from ihPSFs and hPSFs. The interaction network of ihPSF ECM was notably more interconnected and denser than that of hPSF ECM. In addition to the presence of additional collagens in ihPSF ECM compared with hPSF ECM, which were also present in the unsupportive MF1×CD1 ECM, ihPSF ECM contained several laminin chains that were not detected in the hPSF ECM. Laminin has been previously shown to support stem cell maintenance (61, 62) and to be expressed by supportive feeder cells (68), so we speculate that ECM interactions that might be inhibitory to hESC growth, such as those potentially provided by collagens, may be overcome by the presence of key supportive components, such as laminin. Thus, the balance between ECM network properties and molecular composition appears critical for the support of stem cell self-renewal.

hESC-supportive ECM from mouse and human feeders shared many common components not detected in unsupportive hPSF ECM, including collagen XII, collagen I, nidogen-1, fibulin-2, fibulin-5, and collagen III. The ECM molecules laminin 511, which was shown to support hESC growth in a xeno-free medium (61), and collagen IV, which maintained hESC self-renewal only with MEF-conditioned medium (19), were detected in ihPSF and HUES1 ECM but not in CD1 MEF ECM, suggesting that the hESCs can tolerate certain molecules that are not essential for maintenance. Because these molecules were present in supportive ihPSF ECM, this would suggest that, in the absence of conditioned medium, they would not be detrimental as part of a mixed ECM in a chemically defined culture system. Furthermore, ihPSF ECM shared many compositional and network similarities with HUES1 ECM, which suggests that hESCs may secrete all of the ECM components necessary for maintenance of pluripotency if exposed to the “trigger” of a supportive substrate.

Fibronectin, EMILIN-1, tenascin C, fibulin-1, and collagen VI α3 chain were expressed in all types of feeders. ihPSFs have been previously shown to produce a larger amount of fibronectin than hPSFs (30). Indeed, normalized spectrum count data showed that fibronectin was enriched in ihPSF ECM 24-fold over hPSF ECM (Table 1). Because fibronectin is known to support hESC growth in the absence of feeders (6, 8, 9, 61), the lower fibronectin content of hPSF ECM may contribute to its failure to support hESC maintenance. Indeed, an increase or decrease in fibronectin concentration away from an optimal, intermediate concentration has been shown to induce a switch in focal adhesion kinase signaling and promote differentiation of mouse embryonic stem cells (65). Our feeder-free system for culturing hESCs on fibronectin-coated plates means that it is difficult to assess the levels of endogenous fibronectin produced by hESCs. However, we can deduce from immunostaining that once hESCs start undergoing the differentiation process, cells begin to organize fibronectin into fibrillar-like structures. In previous proteomic studies of conditioned medium, a high percentage of proteins identified comprised ECM components (23, 69, 70), such as perlecan, fibronectin, and fibrillin-1, which were also identified in our MS analysis of hESC-derived ECM as well as mouse and human feeder-derived ECMs. One of the aims of this study was to define an ECM substrate that sustains undifferentiated self-renewing hESCs. Exploiting our MS data, we identified and tested a number of ECM proteins as potential culture substrates. Some of these molecules were found to maintain hESCs for three passages, including fibrillin-1 as a single substrate, and perlecan and fibulin-2 in combination with a low, otherwise unsupportive, concentration of fibronectin. Other substrates tested, including tenascin C, collagen VI, biglycan, and versican, did not support hESC self-renewal. Single ECM molecules, such as fibronectin (6, 8), laminin (61), and vitronectin (19), have been used previously as substrates for hESC culture. Our MS analysis of the ECM produced by hESCs cultured on fibronectin identified a number of other ECM components, including fibrillin-2, perlecan, thrombospondin, metalloproteinases, and growth factors. This lends weight to the idea that, even when hESCs are cultured on a single substrate, they produce their own specialized niche that may be involved in regulating pluripotency. Our data suggest that when hESCs are grown on a single substrate, they deposit a complex ECM, and it is likely that the interactions between the ECM components are crucial in providing the supportive niche conducive to continued stem cell self-renewal.

Many proteins comprising or associated with fibrillin microfibrils were identified in our proteomic data sets, including fibrillin-1 and -2, fibulin-2, EMILIN-1, and latent TGF-β-binding protein (LTBP)-1 and -2. Fibrillin-1 was identified in both feeder ECMs and HUES1 ECM and was tested successfully as a substrate for at least short term culture of hESCs. Fibrillin-1 has been shown to mediate cell adhesion via integrin α5β1 (71), which we showed here was expressed by hESCs. Furthermore, fibrillin-1 has been reported to regulate the bioavailability of TGF-β (72), whose role in maintaining pluripotency through Smad pathway activation is well established (67). Indeed, our MS data revealed that ECMs produced by feeder cells and HUES1 cells contained TGF-β as well as LTBP-1 and thrombospondin-1, which are known to activate latent TGF-β (72, 73). Activation of TGF-β is normally tightly regulated, and the effects of TGF-β family signaling on stem cell pluripotency are diverse (74). Because both high and very low concentrations of TGF-β family members can induce hESC differentiation, fibrillin-1, along with appropriate networks of ECM molecules, may function to modulate levels of TGF-β signaling appropriate to control stem cell maintenance or differentiation. Thus, the function of ECM molecules in regulating the availability of growth factors is likely to play a critical role in the maintenance of hESC pluripotency.

Perlecan was identified in both the feeder ECMs and the HUES1 ECM, which is consistent with previous reports analyzing conditioned media and feeder cells (70, 75). Perlecan interacts with fibronectin, fibulin-2, nidogen, and collagen IV, all of which were identified in our proteomic data sets. hESCs cultured on 20 μg/ml perlecan in combination with 5 μg/ml fibronectin retained their Oct4 expression after three passages, which is in agreement with a recently published paper by Abraham *et al.* (75) that also showed that perlecan in combination with fibronectin can support hESC pluripotency. Perlecan binds FGF through its heparan sulfate side chains (76) and promotes FGF receptor binding to modulate angiogenesis (77). Indeed, in endothelial cells, heparan sulfate chains interact with integrins to regulate binding of endostatin, an inhibitor of angiogenesis (78). FGF is an important self-renewal component in routine hESC culture, and it has been shown that heparin in solution can increase hESC survival under certain conditions (79) and that heparin-binding surfaces are supportive of pluripotent hESCs in the long term (80).

Fibulin-2 is an ECM glycoprotein that binds other ECM molecules and, in fibroblasts, is deposited into a meshwork that contains fibronectin (81). In this study, fibulin-2 was identified in ECM produced by mouse feeder cells and HUES1 cells, in accordance with published data (81). Fibulin-2 was able to support maintenance of Oct4-positive hESCs when used in combination with 5 μg/ml fibronectin but not when used as a single substrate. Because fibulin-2 has been shown to colocalize with fibronectin in fibrils deposited by human fibroblasts, it is possible that the interaction of these two molecules may facilitate the correct organization of the ECM needed for hESC attachment and growth. Little is known about the influence of fibulin-2 on stem cell self-renewal, so further work is necessary to determine the role of fibulin-2 in conjunction with fibronectin in supporting pluripotency.

In summary, our proteomic analysis allowed the cataloguing and comparison of ECMs that are supportive and unsupportive for hESC self-renewal. Some ECM proteins were enriched or expressed only in supportive ECM, and we demonstrated that several of these candidates alone or in combination with fibronectin could act as substrates to support at least short term self-renewal of hESCs. Furthermore, the presence of key supportive proteins in native ECMs may be sufficient to permit successful hESC growth, even in the presence of unsupportive components. Indeed, mouse and human feeder cells produced complex networks of ECM proteins with distinct compositions and network topologies, which suggests that the balance between ECM network properties, molecular composition, and specific protein-protein interactions plays a role in the maintenance of pluripotency. Given the outstanding need for a better understanding of stem cell maintenance, our data provide a useful resource for the further study of stem cell growth *in vitro* and microenvironmental control of stem cell function and fate *in vivo*.

## Supplementary Material

Supplemental Data

## Footnotes

10 The abbreviations used are:

hESC: human embryonic stem cell
MEF: mouse embryonic fibroblast
ECM: extracellular matrix
hPSF: human placental stromal fibroblast
ihPSF: immortalized human placental stromal fibroblast
P*n*: passage *n*
LTBP: latent TGF-β-binding protein.
