# Supplementary material for: Comparative Proteomic Analysis of Supportive and Unsupportive Extracellular Matrix Substrates for Human Embryonic Stem Cell Maintenance
Source: J Biol Chem. 2013 May 8;288(26):18716–31. doi: 10.1074/jbc.M113.463372 (PMC3696646; doi:10.1074/jbc.M113.463372)
Supplement: Supplemental Data [file supp_288_26_18716__index.html]

Comparative Proteomic Analysis of Supportive and Unsupportive Extracellular Matrix Substrates for Human Embryonic Stem Cell Maintenance — Proteomic Analysis of ECM Supporting hESC Maintenance — Supplemental Data 

# Comparative Proteomic Analysis of Supportive and Unsupportive Extracellular Matrix Substrates for Human Embryonic Stem Cell Maintenance

## Supplemental Data

**Files in this Data Supplement:**

- Supplemental Data (.pdf, 5.2 MB) - Supplemental Figures S1-S7, Supplemental Tables S1-S4
- Supplemental File S1 (.xlsx, 4.6 MB) - Protein, peptide and spectrum reports for CD1 MEF and MF1xCD1 MEF ECM datasets
- Supplemental File S2 (.xlsx, 1.9 MB) - Protein, peptide and spectrum reports for ihPSF and hPSF ECM datasets
- Supplemental File S3 (.xlsx, 393 KB) - Protein, peptide and spectrum reports for HUES1 ECM dataset
- Supplemental File S4 (.zip, 19 KB) - Interaction network for CD1 MEF ECM
- Supplemental File S5 (.zip, 25 KB) - Interaction network for MF1xCD1 MEF ECM
- Supplemental File S6 (.zip, 17 KB) - Interaction network for ihPSF ECM
- Supplemental File S7 (.zip, 15 KB) - Interaction network for hPSF ECM
- Supplemental File S8 (.zip, 9 KB) - Interaction network for HUES1 ECM
