# Supplementary material for: Comparative Proteomic Analysis of Supportive and Unsupportive Extracellular Matrix Substrates for Human Embryonic Stem Cell Maintenance
Source: J Biol Chem. 2013 May 8;288(26):18716–31. doi: 10.1074/jbc.M113.463372 (PMC3696646; doi:10.1074/jbc.M113.463372)
Supplement: Supplemental Data [file supp_M113.463372_jbc.M113.463372-1.pdf]

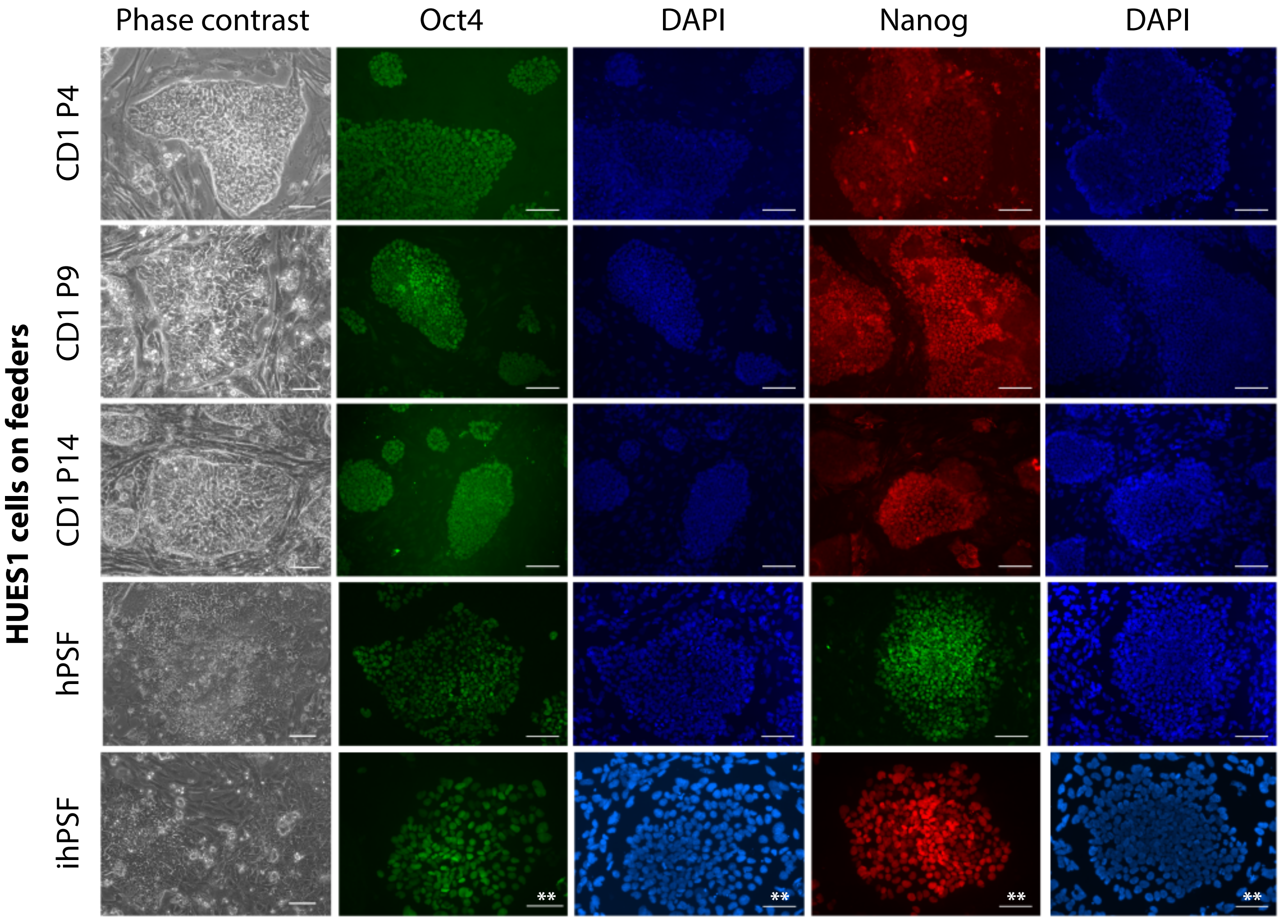

SUPPLEMENTAL FIGURE S1. **Culture of hESCs on mouse and human feeder cells.** HUES1 cells were cultured on CD1 MEFs, hPSFs and ihPSFs. Cells were stained for Oct4 and Nanog; cell nuclei were stained with DAPI. *Scale bars*, 100 µm, except: **, 25 µm.

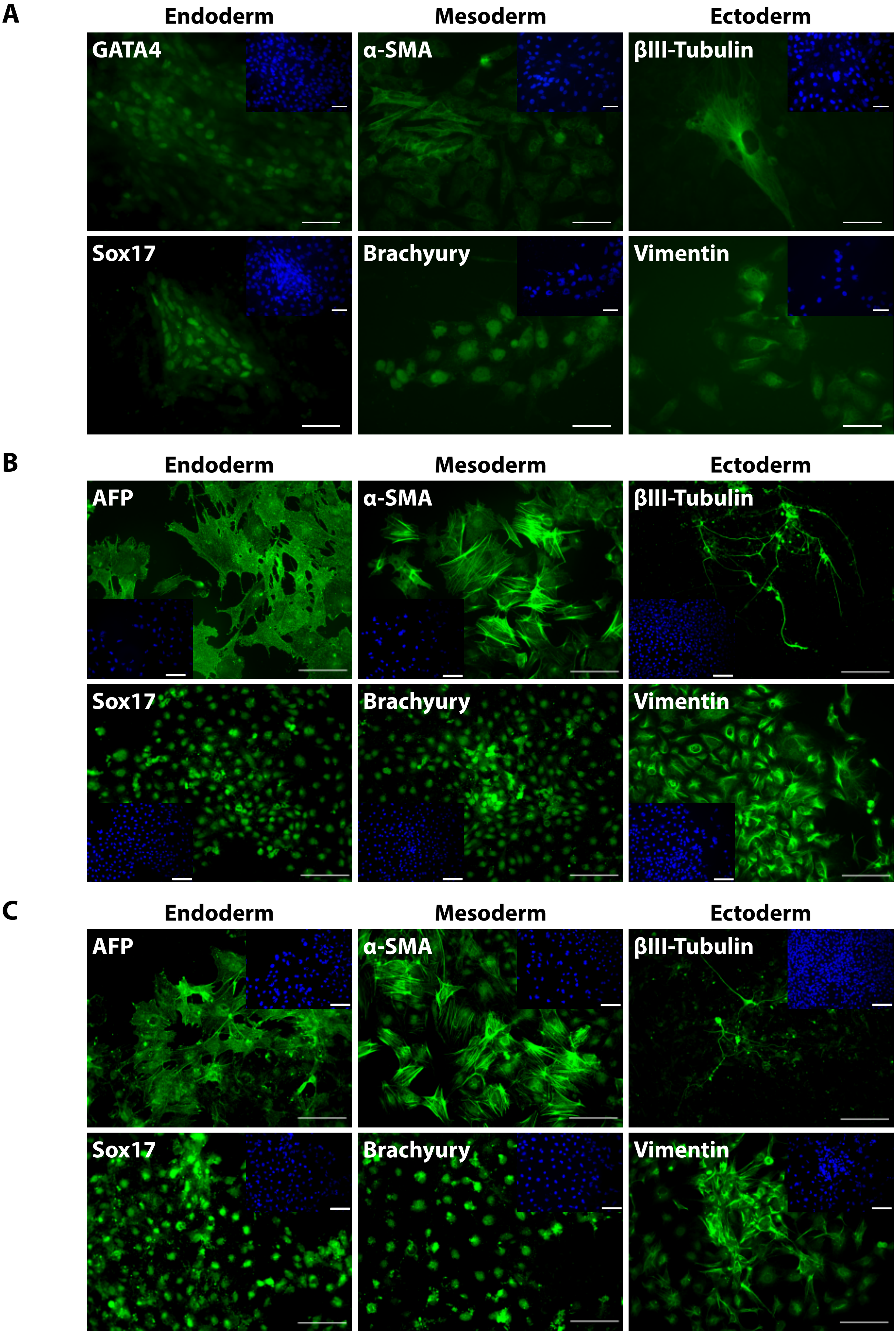

SUPPLEMENTAL FIGURE S2. ***In vitro* differentiation of hESCs.** *A*, day-10 embryoid body outgrowths generated from HUES1 cells cultured for at least five passages on CD1 P4 ECM. *B*, day-10 embryoid body outgrowths generated from HUES1 cells cultured for at least five passages on ihPSF ECM. *C*, day-10 embryoid body outgrowths generated from HUES7 cells cultured for at least five passages on CD1 P4 ECM. Outgrowths were stained (*green*) for endoderm markers GATA4, α-fetoprotein (AFP) and Sox17; mesoderm markers α-smooth muscle actin (α-SMA) and brachyury; and ectoderm markers βIII-tubulin and vimentin. Cell nuclei were stained with DAPI (*blue*; *insets*). *Scale bars*, 50 µm.

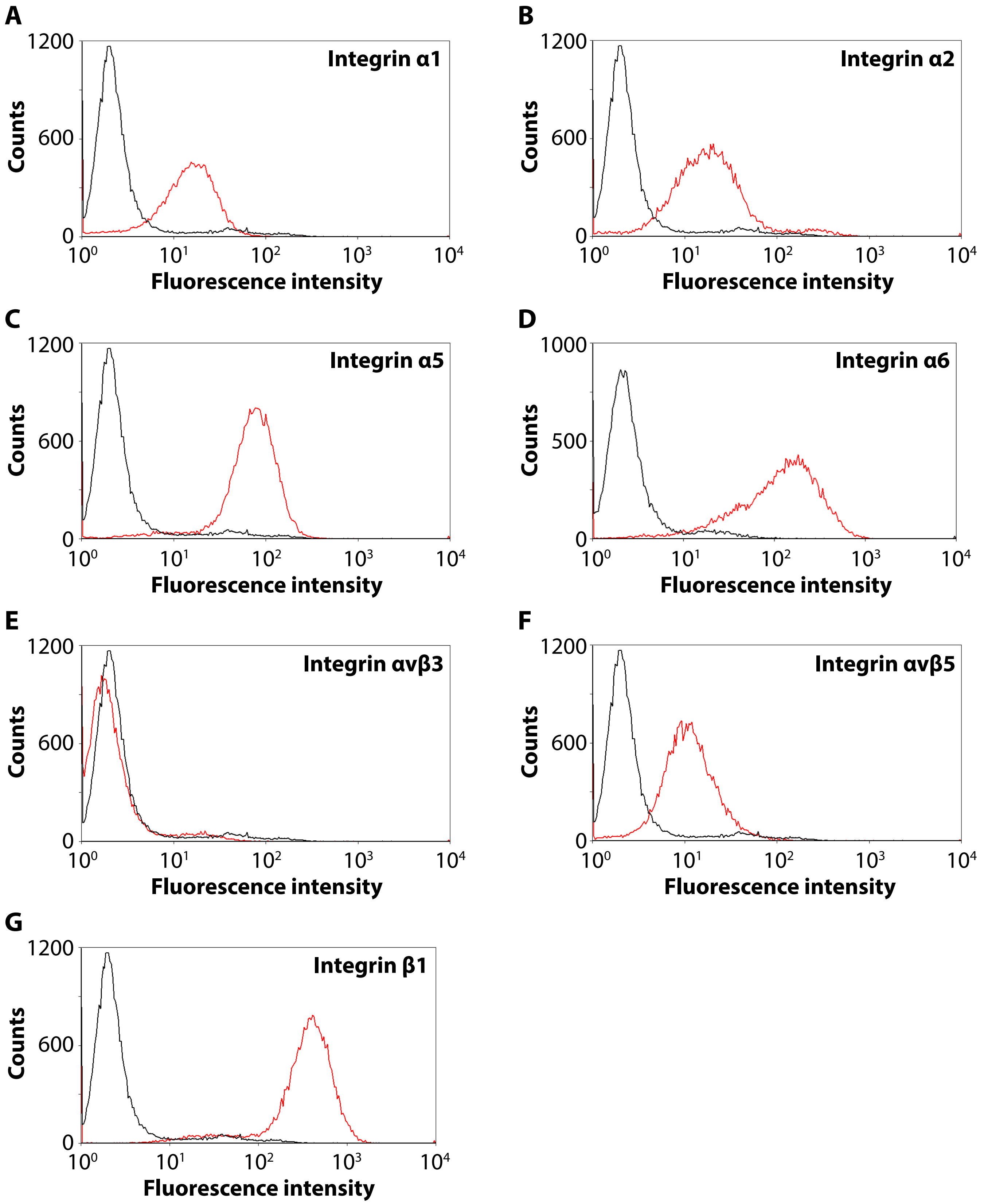

SUPPLEMENTAL FIGURE S3. **Flow cytometric analysis of integrins expressed in hESCs.** *A–G*, HUES1 cells were analyzed by flow cytometry with a panel of integrin receptor antibodies against integrins α1 (*A*), α2 (*B*), α5 (*C*), α6 (*D*), αvβ3 (*E*), αvβ5 (*F*) and β1 (*G*). HUES1 cells cultured on fibronectin expressed all integrin receptors tested except αvβ3 (*E*). Negative IgG controls are represented by *black lines* and the expression of integrins is represented by *red lines*. Fluorescence intensity (*x*-axis) is plotted on a log_{10} scale.

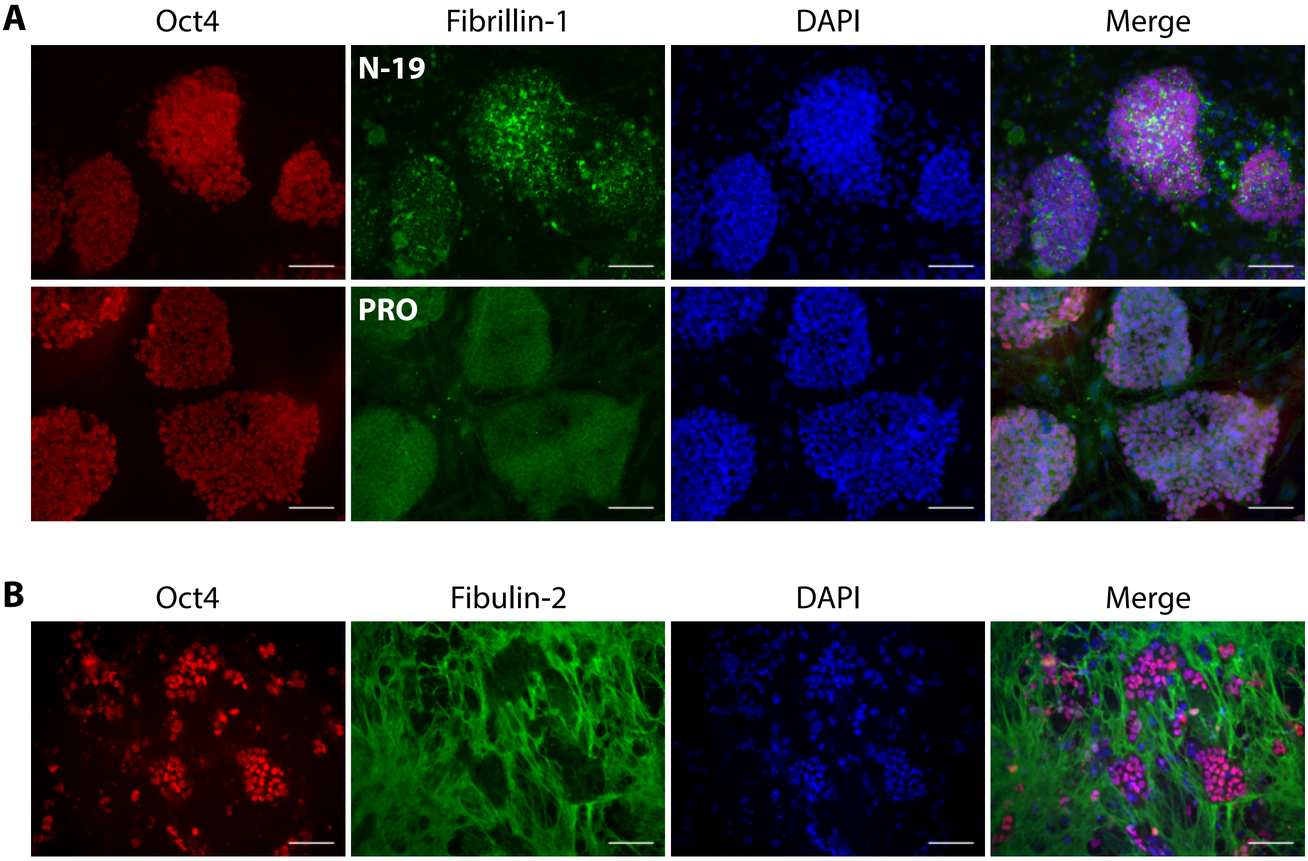

SUPPLEMENTAL FIGURE S4. **ECM protein distribution in hESCs cultured on mouse feeder cells.** *A*, representative immunostaining images showing the distribution of fibrillin-1 (*green*) associated with hESCs cultured on CD1 P4 feeder cells. Fibrillin-1 staining was restricted to hESC colonies. Two anti–fibrillin-1 antibodies were used: N-19 (*top panel*) recognizes the N-terminal region; PRO (*bottom panel*) recognizes the proline-rich region. *B*, distribution of fibulin-2 (*green*) associated with hESCs cultured on CD1 P4 feeder cells. Fibulin-2 formed a fibrillar network that appeared to surround the hESC colonies. HUES1 cells displayed expression of the pluripotency-associated marker Oct4 (*red*). Cell nuclei were stained with DAPI. *Scale bars*, 100 μm.

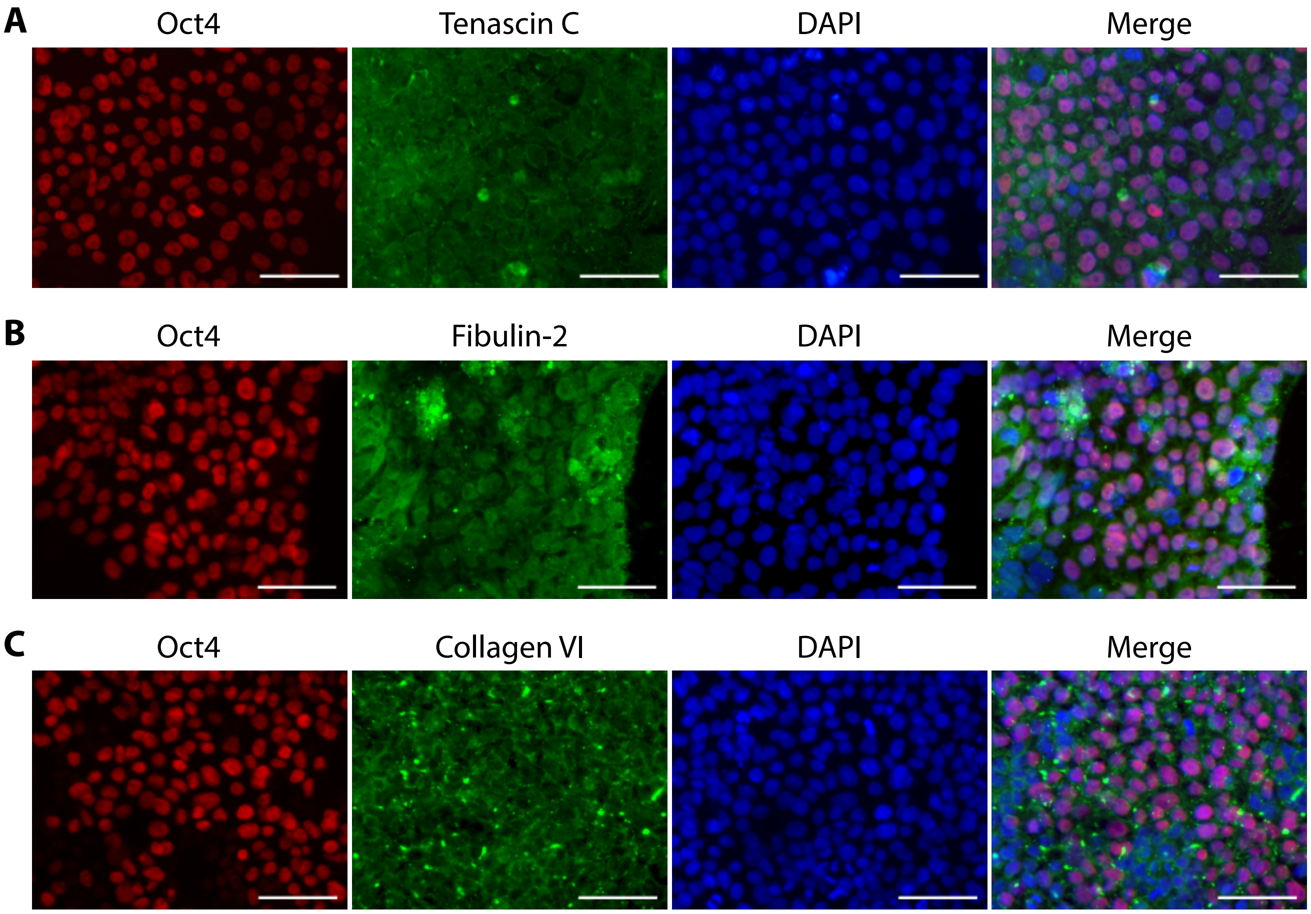

SUPPLEMENTAL FIGURE S5. **ECM protein distribution in hESCs cultured on fibronectin.** *A–C*, representative immunostaining images showing the distribution of tenascin C (*A*; *green*), fibulin-2 (*B*; *green*) and collagen VI (*C*; *green*) associated with hESCs cultured feeder-free on fibronectin. HUES1 cells displayed expression of the pluripotency-associated marker Oct4 (*red*). Cell nuclei were stained with DAPI. *Scale bars*, 100 μm.

A

HUES1 P25: 46,XX,der(17)(q25–>p13::q11.2–>q25)

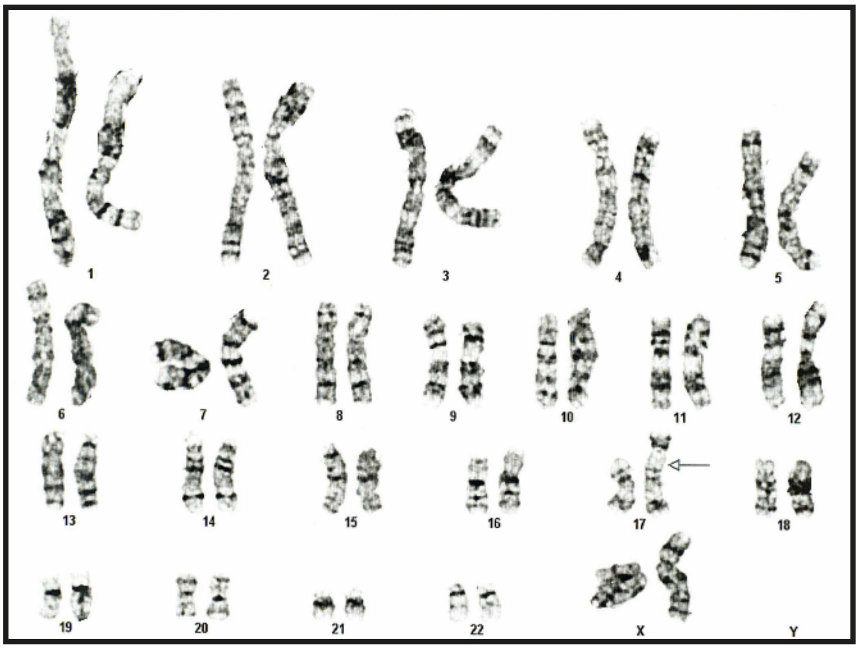

B

HUES7 P36: 46,XY

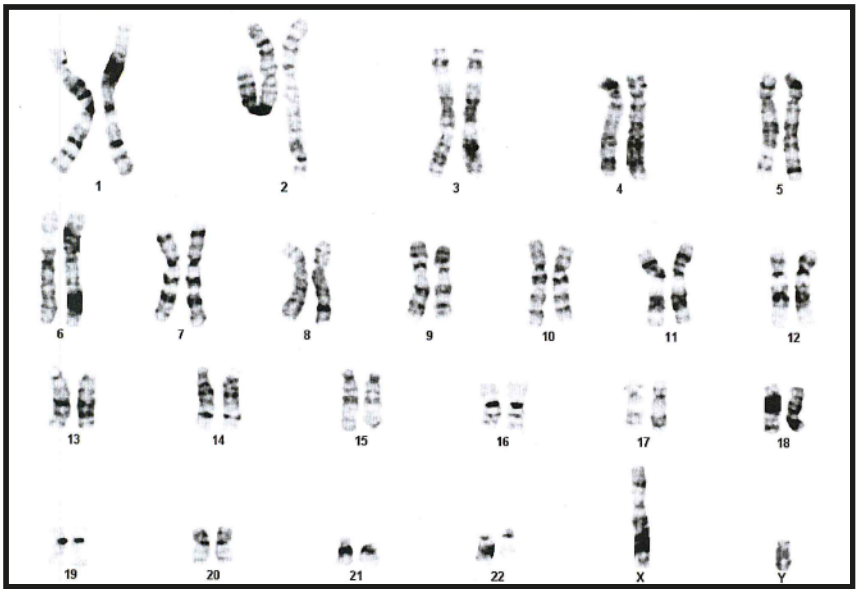

SUPPLEMENTAL FIGURE S6. **Karyotypic analysis of hESCs cultured feeder free.** *A* and *B*, representative G-banded metaphase spreads of HUES1 P25 (A) and HUES7 P36 (B). hESCs were cultured for three passages in feeder-free conditions on fibronectin. HUES1 (A) carries a translocation common to hESC lines (refs 82, 83), with the duplication of the long arm of chromosome 11q translocated to chromosome 17 (*arrow*).

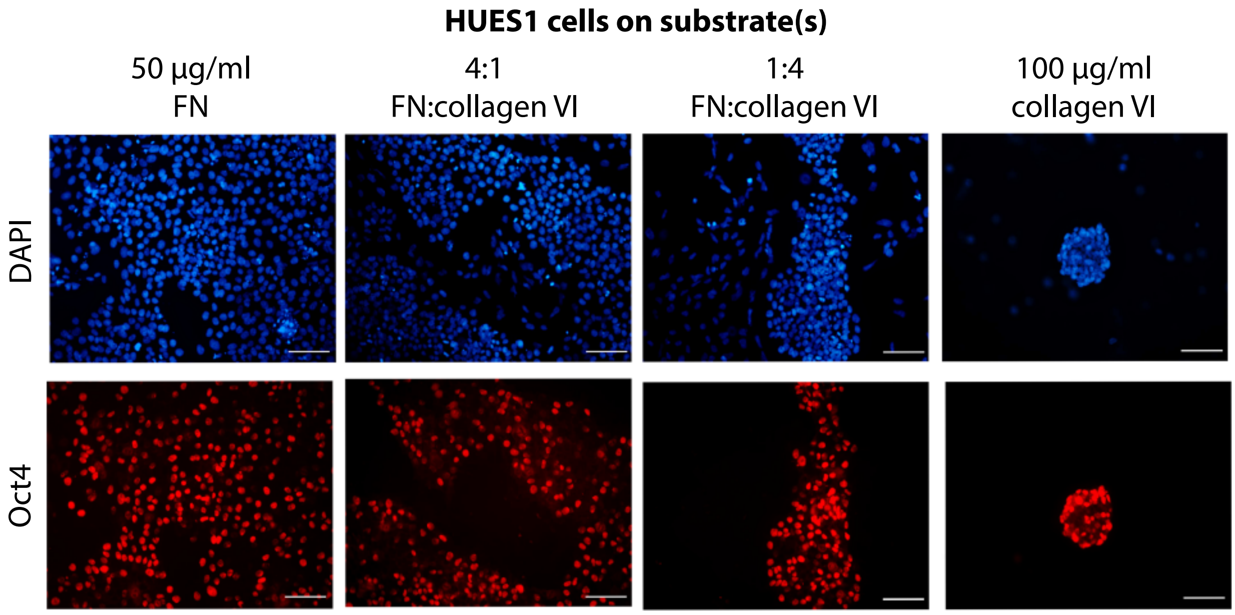

SUPPLEMENTAL FIGURE S7. **The effects of collagen VI on hESCs in the feeder-free culture system.** Culture of HUES1 cells on collagen VI alone resulted in detachment of HUES1 cells and decreased Oct4 expression, although some HUES1 cells retained their pluripotency-associated marker expression after long-term culture. When collagen VI was used in combination with fibronectin (*FN*) as a substrate, the positive effect of fibronectin on cell attachment and pluripotency-associated marker expression was diminished in a dose-dependent manner as the proportion of collagen VI was increased. Cell nuclei were stained with DAPI. *Scale bars*, 100 µm.

SUPPLEMENTAL TABLE S1.

Hierarchical clustering analysis of proteins identified in CD1 and MF1×CD1 ECMs (complete datasets).

Cell-derived ECMs were isolated and analysed by MS as described in "Experimental Procedures." Output of hierarchical clustering is represented in Figure 3.

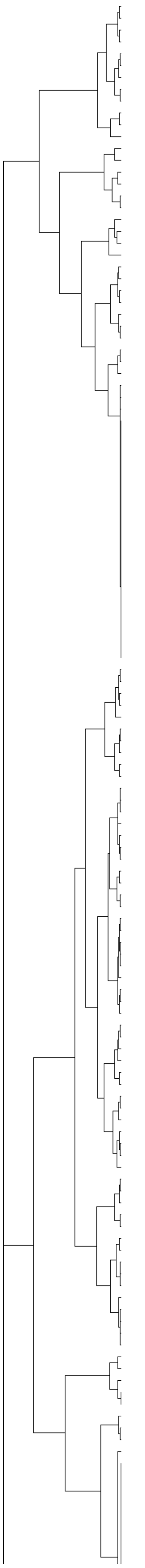

Hierarchical clustering	Mouse gene symbol	Human UniProt Accession	IPI Accession	Protein name	Extracellular?[a]	Cell surface?[a]	Mean normalised spectrum count (% total spectra) ×10³ CD1 P4	CD1 P9	MF1xCD1 P4	MF1xCD1 P9
	Hspg2	P98160	IPI00515360	perlecan	Yes	–	6.63	13.10	10.30	15.83
	Tnc	P24821	IPI00403938	Isoform 1 of Tenascin	Yes	–	1.17	3.21	2.91	4.58
	Emilin1	Q9Y6C2	IPI00115516	EMILIN-1	Yes	–	13.70	27.34	13.00	34.66
	Rpl6	Q02878	IPI00313222	60S ribosomal protein L6	–	–	0.98	2.30	1.20	2.32
	Efemp2	O95967	IPI00126055	EGF-containing fibulin-like extracellular matrix protein 2	Yes	–	16.70	16.35	6.26	31.61
	H2afy	O75367	IPI00137852	Isoform 1 of Core histone macro-H2A.1	–	–	8.40	8.73	2.36	16.89
	Hnrnpu	Q00839	IPI00458583	Heterogeneous nuclear ribonucleoprotein U	–	Yes	0.75	1.02	0	1.80
	Flot2	Q14254	IPI00754549	Isoform 1 of Flotillin-2	–	Yes	3.74	10.10	2.17	10.65
	Hist1h1d	P16402	IPI00331597	Histone H1.3	–	–	7.66	17.31	2.78	16.76
	Myh10	P35580	IPI00515398	Myosin-10	–	Yes	3.41	8.42	0.34	4.30
	Flot1	O75955	IPI00117181	Flotillin-1	–	Yes	4.60	11.69	2.55	6.04
	Rpl35	P42766	IPI00263879	60S ribosomal protein L35	–	–	0	11.47	0	8.78
	Tgfbi	Q15582	IPI00122528	Transforming growth factor-beta-induced protein ig-h3	Yes	–	2.69	4.39	7.36	5.27
	Rps7	P62081	IPI00136984	40S ribosomal protein S7	–	–	0	2.37	4.64	4.79
	Col12a1	Q99715	IPI00121430	Isoform 1 of Collagen alpha-1(XII) chain	Yes	–	2.60	1.91	8.23	6.44
	Gnb1	P62873	IPI00120716	Guanine nucleotide-binding protein G(I)/G(S)/G(T) subunit beta-1	–	Yes	0.90	0	2.08	1.42
	Rpl31	P62899	IPI00123007	60S ribosomal protein L31	–	–	0	0	8.74	7.53
	Itgb1	P05556	IPI00132474	Integrin beta-1	–	Yes	0	0	0.93	0.90
	Lmna	P02545	IPI00620256	Isoform A of Lamin-A/C	Yes	–	17.28	7.79	10.15	40.55
	Fbln2	P98095	IPI00132067	Isoform 1 of Fibulin-2	Yes	–	54.37	31.82	32.76	69.58
	Ltbp2	Q14767	IPI00117380	latent transforming growth factor beta binding protein 2	Yes	–	1.04	0.35	0.21	1.23
	Cd44	P16070	IPI00223769	CD44 antigen isoform c	–	Yes	2.49	0	2.55	3.95
	Htra1	Q92743	IPI00930771	HtrA serine peptidase 1 precursor	Yes	–	5.31	14.07	10.81	40.81
	Col1a1	P02452	IPI00329872	Isoform 1 of Collagen alpha-1(I) chain	Yes	–	0	1.14	1.45	3.70
	Col6a3	P12111	IPI00830749	Col6a3 protein	Yes	–	0.23	0.23	0.48	1.73
	Col1a2	P08123	IPI00222188	Collagen alpha-2(I) chain	Yes	–	0.44	0.52	1.34	3.26
	Col6a1	P12109	IPI00339885	Collagen alpha-1(VI) chain	Yes	–	0	0	0.80	1.46
	Ckap4	Q07065	IPI00223047	Cytoskeleton-associated protein 4	–	–	0	0	0.64	2.06
	Efemp1	Q12805	IPI00223457	EGF-containing fibulin-like extracellular matrix protein 1	Yes	–	0	0	0.74	1.92
	Aqp1	P29972	IPI00123183	Aquaporin-1	–	Yes	0	9.81	0	39.06
	H2afy2	Q9P0M6	IPI00652934	Core histone macro-H2A.2	–	–	0	3.92	0	12.51
	Ltbp1	Q14766	IPI00409393	187 kDa protein	Yes	–	0	0.56	0	1.13
	Adamtsl4	Q6UY14	IPI00279302	ADAMTS-like protein 4	Yes	–	1.05	0	0.54	11.77
	Nid2	Q14112	IPI00719919	nidogen 2 precursor	Yes	–	0.37	0.73	0	15.46
	Fbn1	P35555	IPI00338565	fibrillin 1	Yes	–	0.27	0	0.26	10.07
	Hist1h1e	P10412	IPI00223714	Histone H1.4	–	–	0	0	0	17.96
	Mmp14	P50281	IPI00133725	Matrix metalloproteinase-14	Yes	–	0	0	0	2.00
	Fbn2	P35556	IPI00272591	fibrillin 2 precursor	Yes	–	0	0	0	0.67
	Npnt	Q6UXI9	IPI00124689	Isoform 4 of Nephronectin	Yes	–	0	0	0	4.72
	Nt5e	P21589	IPI00122257	5'-nucleotidase	–	Yes	0	0	0	1.65
	Cacna2d1	P54289	IPI00230013	Isoform 2B of Voltage-dependent calcium channel subunit alpha-2/delta-1	–	Yes	0	0	0	0.43
	Vcam1	P19320	IPI00126834	Isoform 1 of Vascular cell adhesion protein 1	–	Yes	0	0	0	0.65
	Emilin2	Q9BXX0	IPI00284848	EMILIN-2	Yes	–	0	0	0	1.25
	Hist1h1b	P16401	IPI00230133	Histone H1.5	–	–	0	0	0	3.44
	Col6a2	P12110	IPI00621027	Collagen alpha-2(VI) chain	Yes	–	0	0	0	0.96
	Cltc	Q00610	IPI00169916	Clathrin heavy chain 1	–	Yes	0	0	0	0.41
	Pxdn	Q92626	IPI00461384	Peroxidasin homolog	Yes	–	0	0	0	0.48
	Rpl8	P62917	IPI00137787	60S ribosomal protein L8	–	–	0	0	0	1.88
	Sun1	O94901	IPI00227435	Isoform 3 of Protein unc-84 homolog A	–	–	0	0	0	0.81
	Gnas	Q5JWF2	IPI00416906	Isoform XLas-1 of Guanine nucleotide-binding protein G(s) subunit alpha isoforms XLas	–	Yes	0	0	0	1.30
	Cenpv	Q7Z7K6	IPI00318428	Isoform 1 of Centromere protein V	–	–	0	0	0	2.82
	Sun2	Q9UH99	IPI00380009	Protein unc-84 homolog B	–	–	0	0	0	0.68
	Lmnb1	P20700	IPI00230394	Lamin-B1	–	–	0	0	0	3.15
	Naglu	P54802	IPI00314726	alpha-N-acetylglucosaminidase	–	–	0	0	0	0.63
	Krt31	Q15323	IPI00124945	Keratin, type I cuticular Ha1	–	–	0	0	0	3.10
	Krt85	P78386	IPI00331459	Keratin, type II cuticular Hb5	–	–	0	0	0	2.60
	Fn1	P02751	IPI00652813	Putative uncharacterized protein	Yes	–	99.09	116.59	95.16	118.09
	Thy1	P04216	IPI00109727	Thy-1 membrane glycoprotein	–	Yes	20.04	22.01	19.60	20.49
	Krt1	P04264	IPI00625729	Keratin, type II cytoskeletal 1	–	Yes	8.78	12.91	6.65	11.58
	Gnai2	P04899	IPI00652902	Putative uncharacterized protein	–	Yes	6.05	8.78	5.63	6.59
	Rpl23a	P62750	IPI00461456	60S ribosomal protein L23a	–	–	13.19	16.41	6.80	8.78
	Thbs1	P07996	IPI00118413	Thrombospondin 1	Yes	–	13.85	28.96	20.78	7.84
	Postn	Q15063	IPI00338018	Isoform 1 of Periostin	Yes	–	7.95	21.17	15.72	3.97
	Rps2	P15880	IPI00380130	Putative uncharacterized protein	–	–	2.65	9.72	6.15	3.16
	Krt10	P13645	IPI00755181	keratin complex 1, acidic, gene 10	–	–	5.54	14.63	6.81	7.39
	Rps9	P46781	IPI00420726	40S ribosomal protein S9	–	–	4.32	10.57	7.12	6.87
	Hist1h4a	P62805	IPI00329998	11 kDa protein	–	–	371.96	296.81	268.18	234.70
	Hist1h2bf	P62807	IPI00114642	Histone H2B type 1-F/J/L	–	–	138.61	116.28	109.56	101.61
	Rpl24	P83731	IPI00323806	Putative uncharacterized protein	–	–	21.48	14.51	13.60	14.64
	Vim	P08670	IPI00227299	Vimentin	–	Yes	68.70	59.21	74.91	47.27
	Myl6	P60660	IPI00354819	Isoform Smooth muscle of Myosin light polypeptide 6	–	–	92.81	71.24	67.94	43.85
	Eef1a1	P68104	IPI00307837	Elongation factor 1-alpha 1	–	–	7.40	4.48	5.52	2.63
	Rpl17	P18621	IPI00453768	60S ribosomal protein L17	–	–	10.74	6.22	8.28	5.02
	Serpine1	P05121	IPI00131547	Plasminogen activator inhibitor 1	Yes	–	15.24	5.98	15.32	3.51
	Loxl1	Q08397	IPI00380136	Lysyl oxidase homolog 1	Yes	–	6.73	3.24	7.01	2.75
	Actc1	P68032	IPI00114593	Actin, alpha cardiac muscle 1	Yes	–	179.41	93.88	171.08	106.76
	Vdac2	P45880	IPI00122547	Voltage-dependent anion-selective channel protein 2	–	–	6.67	2.81	7.03	4.12
	Rps16	P62249	IPI00469918	Rps16 protein	–	–	36.68	33.88	42.27	5.55
	Tubb5	P07437	IPI00117352	Tubulin beta-5 chain	–	–	14.42	14.95	14.43	2.11
	Rps4x	P62701	IPI00331092	40S ribosomal protein S4, X isoform	–	–	24.67	19.33	22.15	6.15
	Tuba1c	Q9BQE3	IPI00403810	Tubulin alpha-1C chain	–	–	10.35	9.80	10.24	2.63
	Rpl11	P62913	IPI00331461	60S ribosomal protein L11	–	–	16.25	14.41	17.64	5.27
	Slc25a4	P12235	IPI00115564	ADP/ATP translocase 1	–	Yes	7.34	6.68	9.76	2.39
	Rps18	P62269	IPI00317590	40S ribosomal protein S18	–	–	54.97	56.93	62.48	23.42
	Hbb-b2	P68871	IPI00316491	Hemoglobin subunit beta-2	–	–	15.13	19.64	19.16	8.23
	Rps14	P62263	IPI00322562	40S ribosomal protein S14	–	–	23.59	30.61	26.54	8.23
	Myh9	P35579	IPI00123181	Myosin-9	–	Yes	11.52	8.28	5.41	6.45
	Rps27a	P62979	IPI00470152	ribosomal protein S27a	–	–	64.63	46.32	32.07	42.44
	Gsn	P06396	IPI00117167	Isoform 1 of Gelsolin	Yes	–	1.93	0.91	0.95	0.92
	Hist1h3b	P68431	IPI00282848	histone cluster 2, H3c1	–	–	22.78	17.02	12.02	7.90
	Fbln5	Q9UBX5	IPI00323035	Fibulin-5	Yes	–	9.06	5.31	1.63	2.63
	Krt2	P35908	IPI00622240	Keratin, type II cytoskeletal 2 epidermal	–	–	2.41	1.27	0.86	0.74
	Actb	P60709	IPI00110850	Actin, cytoplasmic 1	–	–	182.83	113.45	180.82	43.91
	Rps11	P62280	IPI00117569	Putative uncharacterized protein	–	–	29.36	17.72	24.22	4.16
	Rpl23	P62829	IPI00139780	60S ribosomal protein L23	–	–	24.18	11.23	19.04	0
	Rps13	P62277	IPI00125901	40S ribosomal protein S13	–	–	41.16	29.50	24.37	0
	Myo1c	O00159	IPI00620222	Isoform 1 of Myosin-Ic	–	Yes	3.53	3.16	2.73	0
	Mgp	P08493	IPI00117985	Matrix Gla protein	Yes	–	37.75	32.64	23.80	0
	Atp6v0d1	P61421	IPI00313841	V-type proton ATPase subunit d 1	–	Yes	4.21	2.25	1.92	0
	Fbln1	P23142	IPI00322748	Isoform D of Fibulin-1	Yes	–	6.90	7.43	2.29	0
	Lox	P28300	IPI00310056	Protein-lysine 6-oxidase	Yes	–	3.64	3.74	1.74	0
	Rpl13	P26373	IPI00224505	60S ribosomal protein L13	–	–	4.84	3.75	1.70	0
	Myadm	Q96S97	IPI00132938	Myeloid-associated differentiation marker	–	Yes	6.52	6.71	0	0
	Tgfb2	P61812	IPI00114180	Transforming growth factor beta-2	Yes	–	3.16	2.81	0	0
	Prss23	O95084	IPI00318017	Serine protease 23	Yes	–	9.22	14.62	11.77	0
	Rps19	P39019	IPI00113241	40S ribosomal protein S19	–	–	7.25	15.42	9.26	0
	Rps3	P23396	IPI00134599	40S ribosomal protein S3	–	–	31.81	34.22	32.19	0
	Arpc4	P59998	IPI00138691	Actin-related protein 2/3 complex subunit 4	–	–	6.17	7.29	6.65	0
	Hba	P69905	IPI00110658	Putative uncharacterized protein	–	–	18.53	20.95	16.36	0
	Rps3a	P61247	IPI00331345	40S ribosomal protein S3a	–	–	17.55	23.32	12.45	0
	Tpm1	P09493	IPI00227835	Isoform 2 of Tropomyosin alpha-1 chain	–	Yes	2.51	3.96	1.24	0
	Cyr61	O00622	IPI00113117	Protein CYR61	Yes	–	1.58	2.68	0.73	0
	Lgals9	O00182	IPI00114396	Isoform Long of Galectin-9	Yes	–	1.24	2.25	0.77	0
	Ran	P62826	IPI00134621	GTP-binding nuclear protein Ran	–	–	2.38	4.35	1.70	0
	Rpl27	P61353	IPI00122421	60S ribosomal protein L27	–	–	3.57	9.79	11.47	0
	Rpl9	P32969	IPI00122413	60S ribosomal protein L9	–	–	0	5.12	6.49	0
	Tinagl1	Q9GZM7	IPI00115458	Tubulointerstitial nephritis antigen-like	Yes	–	5.92	25.48	14.39	0
	Rac1	P63000	IPI00127408	RAS-related C3 botulinum substrate 1, isoform CRA_a	–	Yes	0	1.96	1.34	0
	Rps17	P08708	IPI00465880	40S ribosomal protein S17	–	–	0	4.22	2.88	0
	Tgfb3	P10600	IPI00135633	transforming growth factor, beta 3	Yes	–	2.82	4.62	0	0
	Rps8	P62241	IPI00274175	similar to ribosomal protein S8 isoform 1	–	–	1.38	3.26	0	0
	Ptx3	P26022	IPI00114358	Pentraxin-related protein PTX3	Yes	–	2.04	5.42	0	0
	Wisp2	O76076	IPI00129116	WNT1-inducible-signaling pathway protein 2	Yes	–	0	7.77	1.71	0
	Rplp0	P05388	IPI00314950	60S acidic ribosomal protein P0	–	–	0	2.65	0	0
	H1f0	P07305	IPI00404590	Putative uncharacterized protein	–	–	0	3.55	0	0
	Cxcl12	P48061	IPI00108061	Isoform Alpha of Stromal cell-derived factor 1	Yes	–	0	34.75	0	0
	Krt6b	P04259	IPI00131366	Keratin, type II cytoskeletal 6B	–	–	0	7.40	0	0
	Rai14	Q9P0K7	IPI00453820	Ankycorbin	–	–	0	0.48	0	0
	Arpc3	O15145	IPI00124829	Actin-related protein 2/3 complex subunit 3	–	–	0	4.29	0	0
	Hnrnpc	P07910	IPI00130343	Putative uncharacterized protein	–	–	0	1.22	0	0
	Pcolce	Q15113	IPI00120176	Procollagen C-endopeptidase enhancer 1	Yes	–	0	0.90	0	0
	Tcp1	P17987	IPI00459493	Isoform 1 of T-complex protein 1 subunit alpha B	–	Yes	0	2.18	0	0

Hierarchical clustering

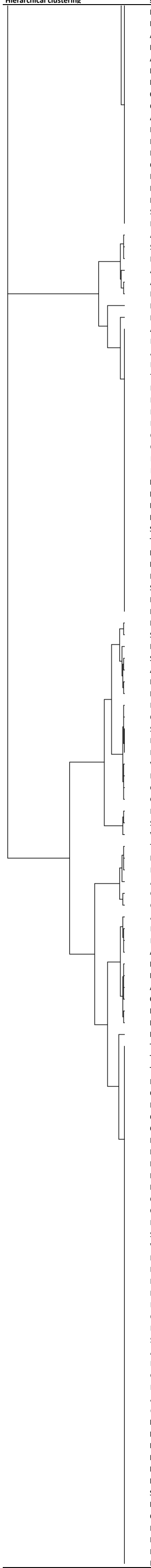

Mouse gene symbol	Human UniProt Accession	IPI Accession	Protein name	Extracellular?[a]	Cell surface?[a]	Mean normalised spectrum count (% total spectra) $\times 10^3$			
						CD1 P4	CD1 P9	MF1xCD1 P4	MF1xCD1 P9
Rpl13a	P40429	IPI00223217	60S ribosomal protein L13a	–	–	0	2.27	0	0
Islr	O14498	IPI00123635	Immunoglobulin superfamily containing leucine-rich repeat protein	Yes	–	0	0.98	0	0
Adamts4	O75173	IPI00469188	A disintegrin and metalloproteinase with thrombospondin motifs 4	Yes	–	0	0.50	0	0
Hnrnpul2	Q1KMD3	IPI00849047	similar to heterogeneous nuclear ribonucleoprotein U-like 2	–	–	0	0.53	0	0
Arpc2	O15144	IPI00661414	Actin-related protein 2/3 complex subunit 2	–	Yes	0	1.32	0	0
Ddx5	P17844	IPI00420363	Probable ATP-dependent RNA helicase DDX5	–	–	0	1.31	0	0
Lgals8	O00214	IPI00408061	Putative uncharacterized protein (Fragment)	Yes	–	0	1.22	0	0
Cct8	P50990	IPI00469268	T-complex protein 1 subunit theta	–	–	0	1.31	0	0
Capzb	P47756	IPI00269481	Isoform 2 of F-actin-capping protein subunit beta	–	–	0	1.45	0	0
Arpc1b	O15143	IPI00125143	Arpc1b protein	–	–	0	1.91	0	0
Rpl14	P50914	IPI00889222	hypothetical protein	–	–	0	3.26	0	0
Itih2	P19823	IPI00227834	inter-alpha trypsin inhibitor, heavy chain 2	Yes	–	0	0.42	0	0
Mfap2	P55001	IPI00135290	microfibril-associated glycoprotein 2	Yes	–	0	2.14	0	0
Col3a1	P02461	IPI00129571	Collagen alpha-1(III) chain	Yes	–	0	0.49	0	0
Hspg2	P98160	IPI00113824	Basement membrane-specific heparan sulfate proteoglycan core protein	Yes	–	0	8.48	0	0
Igsf10	Q6WRI0	IPI00405810	Immunoglobulin superfamily member 10	Yes	–	0	0.16	0	0
Hnrnpm	P52272	IPI00132443	Isoform 1 of Heterogeneous nuclear ribonucleoprotein M	–	Yes	0	1.00	0	0
Sfrp1	Q8N474	IPI00116407	Secreted frizzled-related protein 1	Yes	–	0	2.57	0	0
Ncl	P19338	IPI00317794	Nucleolin	–	Yes	0	0.58	0	0
Atp5b	P06576	IPI00468481	ATP synthase subunit beta, mitochondrial	–	Yes	10.68	0.80	4.57	0
S100a4	P26447	IPI00124096	Protein S100-A4	–	–	9.67	0	5.13	0
Hspa9	P38646	IPI00133903	Stress-70 protein, mitochondrial	–	Yes	2.28	0	1.04	0
Atp5a1	P25705	IPI00130280	ATP synthase subunit alpha, mitochondrial	–	Yes	7.44	1.50	2.73	0
Angptl2	Q9UKU9	IPI00126864	Angiopoietin-related protein 2	Yes	–	4.36	0	1.07	0
Hspd1	P10809	IPI00308885	Isoform 1 of 60 kDa heat shock protein, mitochondrial	Yes	–	2.29	0	0.76	0
Rps26	P62854	IPI00261455	similar to ribosomal protein S26	–	–	11.48	0	0	6.08
Rps29	P62273	IPI00222553	40S ribosomal protein S29	–	–	28.80	7.46	0	0
Atp5o	P48047	IPI00118986	ATP synthase subunit O, mitochondrial	–	Yes	8.05	0	0	0
Phb	P35232	IPI00133440	Prohibitin	–	Yes	3.96	0	0	0
Atp6v0a1	Q93050	IPI00130187	Isoform A1-II of V-type proton ATPase 116 kDa subunit a isoform 1	–	Yes	0.68	0	0	0
Lgals3bp	Q08380	IPI00119809	Galectin-3-binding protein	Yes	–	1.81	0	0	0
Tmed10	P49755	IPI00466570	Isoform 1 of Transmembrane emp24 domain-containing protein 10	–	–	1.99	0	0	0
Lrrc59	Q96AG4	IPI00123281	Leucine-rich repeat-containing protein 59	–	–	0.95	0	0	0
Hspb1	P04792	IPI00128522	Isoform A of Heat shock protein beta-1	–	Yes	5.77	0	0	0
Hmga2	P52926	IPI00331612	High mobility group protein HMGI-C	–	–	8.29	0	0	0
Etfb	P38117	IPI00121440	Electron transfer flavoprotein subunit beta	–	–	6.61	0	0	0
Chchd3	Q9NX63	IPI00133562	Coiled-coil-helix-coiled-coil-helix domain-containing protein 3, mitochondrial	–	–	1.91	0	0	0
Cnn1	P51911	IPI00116645	Isoform Alpha of Calponin-1	–	–	1.00	0	0	0
Rpn2	P04844	IPI00475154	Dolichyl-diphosphooligosaccharide--protein glycosyltransferase subunit 2	–	–	1.20	0	0	0
Rps20	P60866	IPI00323819	40S ribosomal protein S20	–	–	3.83	0	0	0
Rpl30	P62888	IPI00222549	60S ribosomal protein L30	–	–	2.55	0	0	0
Rpl12	P30050	IPI00849793	60S ribosomal protein L12	–	–	1.84	0	0	0
Dad1	P61803	IPI00109082	Dolichyl-diphosphooligosaccharide--protein glycosyltransferase subunit DAD1	–	–	4.14	0	0	0
Myl9	P24844	IPI00750595	Myosin regulatory light polypeptide 9	–	–	16.58	0	0	0
S100a11	P31949	IPI00119202	Protein S100-A11	–	Yes	6.03	0	0	0
Timp3	P35625	IPI00110370	Metalloproteinase inhibitor 3	Yes	–	3.57	0	0	0
Rpn1	P04843	IPI00309035	Dolichyl-diphosphooligosaccharide--protein glycosyltransferase subunit 1	–	–	0.48	0	0	0
Etfa	P13804	IPI00116753	Electron transfer flavoprotein subunit alpha, mitochondrial	–	–	1.42	0	0	0
Ddost	P39656	IPI00117705	Dolichyl-diphosphooligosaccharide--protein glycosyltransferase 48 kDa subunit	–	–	1.75	0	0	0
Sec61b	P60468	IPI00133030	Protein transport protein Sec61 subunit beta	–	–	3.32	0	0	0
Prkcdbp	Q969G5	IPI00126939	Protein kinase C delta-binding protein	–	–	1.18	0	0	0
Lima1	Q9UHB6	IPI00112339	LIM domain and actin binding 1 isoform a	–	Yes	0.59	0	0	0
Flna	P21333	IPI00131138	Isoform 1 of Filamin-A	–	Yes	0.94	0	1.60	0.28
Serpinh1	P50454	IPI00114733	Serpin H1	–	–	5.09	0	8.71	1.68
Rpl10	P27635	IPI00474637	60S ribosomal protein L10	–	–	11.49	3.60	14.70	0
S100a6	P06703	IPI00121427	Protein S100-A6	–	Yes	8.29	0	9.23	0
Actr3	P61158	IPI00115627	Actin-related protein 3	–	Yes	1.06	0	1.31	0
Ehd2	Q9NZN4	IPI00402968	EH domain-containing protein 2	–	Yes	1.36	0	2.01	0
Hsp90ab1	P08238	IPI00229080	MCG18238	–	–	0.40	0	0.56	0
Rpl38	P63173	IPI00138302	similar to ribosomal protein L38	–	–	26.94	0	21.69	0
Cav1	Q03135	IPI00117829	Isoform Alpha of Caveolin-1	–	Yes	8.68	0	7.32	0
Slc25a3	Q00325	IPI00124771	Phosphate carrier protein, mitochondrial	–	Yes	2.90	0	2.69	0
Phb2	Q99623	IPI00321718	Prohibitin-2	–	–	1.00	0	0.93	0
Rps5	P46782	IPI00857345	20 kDa protein	–	–	1.66	0	1.54	0
Vdac1	P21796	IPI00122549	Isoform Pl-VDAC1 of Voltage-dependent anion-selective channel protein 1	–	Yes	5.18	0	3.84	0
Banf1	O75531	IPI00119959	Barrier-to-autointegration factor	–	–	5.70	0	4.08	0
Gpc4	O75487	IPI00312407	Glypican-4	Yes	–	2.86	0	1.94	0
Gng12	Q9UBI6	IPI00227838	Guanine nucleotide-binding protein G(I)/G(S)/G(O) subunit gamma-12	–	Yes	8.29	0	5.77	0
Rps28	P62857	IPI00137736	40S ribosomal protein S28	–	–	12.43	0	21.07	9.88
Slc25a5	P05141	IPI00127841	ADP/ATP translocase 2	–	Yes	4.52	0	7.12	3.99
Vdac3	Q9Y277	IPI00122548	Voltage-dependent anion channel 3	–	–	3.21	0	3.97	2.55
Tgm2	P21980	IPI00126861	Protein-glutamine gamma-glutamyltransferase 2	Yes	–	8.05	6.22	12.35	0
Rps27	P42677	IPI00173160	40S ribosomal protein S27	–	–	14.74	10.01	19.91	0
Lgals1	P09382	IPI00229517	Galectin-1	Yes	–	8.84	6.00	12.62	0
Arf4	P18085	IPI00876029	20 kDa protein	–	–	4.14	2.61	8.19	0
Gapdh	P04406	IPI00273646	Glyceraldehyde-3-phosphate dehydrogenase	–	–	5.53	6.33	15.22	0
Ctgf	P29279	IPI00322594	Connective tissue growth factor	Yes	–	0.87	1.18	2.02	0
Actn1	P12814	IPI00380436	Alpha-actinin-1	–	Yes	2.41	0	9.22	1.28
Des	P17661	IPI00130102	Desmin	–	–	16.58	0	65.47	0
Rab10	P61026	IPI00130118	Ras-related protein Rab-10	–	Yes	3.60	0	17.58	0
Anxa2	P07355	IPI00468203	Annexin A2	Yes	–	1.70	0	7.61	0
Prelp	P51888	IPI00122293	Prolargin	Yes	–	0.77	0	2.50	0
Rps15a	P62244	IPI00230660	40S ribosomal protein S15a	–	–	4.42	0	11.93	0
Anxa1	P04083	IPI00230395	Annexin A1	Yes	–	0.85	0	2.23	0
Cfl1	P23528	IPI00848816	similar to Cofilin-1	–	–	1.75	0	4.86	0
Igf2	P01344	IPI00138397	Insulin-like growth factor II	Yes	–	4.97	0	10.20	0
Rab5c	P51148	IPI00224518	Ras-related protein Rab-5C	–	Yes	1.44	0	3.34	0
Rps24	P62847	IPI00402981	Isoform 2 of 40S ribosomal protein S24	–	–	0	3.00	9.52	0
Thbs2	P35442	IPI00131223	Thrombospondin-2	Yes	–	0	0	1.18	0
Tagln	Q01995	IPI00226515	Transgelin	–	–	0	0	8.01	0
Tpi1	P60174	IPI00467833	triosephosphate isomerase 1	–	–	0	0	4.81	0
Krt14	P02533	IPI00227140	Keratin, type I cytoskeletal 14	–	–	0	0	3.19	0
Clic1	O00299	IPI00130344	Chloride intracellular channel protein 1	–	Yes	0	0	2.28	0
Loxl2	Q9Y4K0	IPI00224208	Isoform 1 of Lysyl oxidase homolog 2	Yes	–	0	0	0.82	0
Cyb5r3	P00387	IPI00110885	Putative uncharacterized protein	–	–	0	0	2.64	0
Col5a1	P20908	IPI00128689	Collagen alpha-1(V) chain	Yes	–	0	0	0.33	0
Ldha	P00338	IPI00319994	L-lactate dehydrogenase A chain	–	–	0	0	2.56	0
Rras2	P62070	IPI00323822	Ras-related protein R-Ras2	–	Yes	0	0	5.34	0
Pfn1	P07737	IPI00224740	Profilin-1	–	–	0	0	6.15	0
Eef2	P13639	IPI00466069	Elongation factor 2	–	–	0	0	0.49	0
Eno1	P06733	IPI00462072	Alpha-enolase	–	Yes	0	0	2.62	0
Cryab	P02511	IPI00138274	Alpha-crystallin B chain	–	–	0	0	8.46	0
Col5a2	P05997	IPI00121120	Collagen alpha-2(V) chain	Yes	–	0	0	0.64	0
Nid1	P14543	IPI00111793	Nidogen-1	Yes	–	0	0	1.08	0
Stom	P27105	IPI00323748	Erythrocyte band 7 integral membrane protein	–	Yes	0	0	4.12	0
Vwa1	Q6PCB0	IPI00331609	Isoform 1 of von Willebrand factor A domain-containing protein 1	Yes	–	0	0	0.91	0
Bgn	P21810	IPI00123194	Biglycan	Yes	–	0	0	6.59	0
Ppia	P62937	IPI00554989	Peptidyl-prolyl cis-trans isomerase	Yes	–	0	0	4.27	0
Hadha	P40939	IPI00223092	Trifunctional enzyme subunit alpha, mitochondrial	–	–	0	0	0.74	0
Rab18	Q9NP72	IPI00116770	Ras-related protein Rab-18	–	Yes	0	0	1.34	0
Itga5	P08648	IPI00115976	Integrin alpha-5	–	Yes	0	0	1.82	0
Col4a2	P08572	IPI00338452	Collagen alpha-2(IV) chain	Yes	–	0	0	0.18	0
Rab7a	P51149	IPI00408892	Ras-related protein Rab-7a	–	–	0	0	1.34	0
Slc25a1	P53007	IPI00276926	solute carrier family 25, member 1	–	–	0	0	0.90	0
Atp5c1	P36542	IPI00313475	ATP synthase subunit gamma, mitochondrial	–	–	0	0	0.93	0
Rab11a	P62491	IPI00323897	Ras-related protein Rab-11A	–	Yes	0	0	2.56	0
Glipr2	Q9H4G4	IPI00416279	Golgi-associated plant pathogenesis-related protein 1	Yes	–	0	0	1.81	0
Rpl7	P18124	IPI00775948	Protein	–	–	0	0	0.93	0
Actn4	O43707	IPI00118899	Alpha-actinin-4	Yes	–	0	0	4.47	0
Csrp1	P21291	IPI00123891	Cysteine and glycine-rich protein 1	–	–	0	0	2.93	0
Pgk1	P00558	IPI00555069	Phosphoglycerate kinase 1	–	–	0	0	1.03	0
Hspa8	P11142	IPI00323357	Heat shock cognate 71 kDa protein	–	Yes	0	0	0.57	0
Ehd1	Q9H4M9	IPI00126083	EH domain-containing protein 1	–	Yes	0	0	1.17	0
Mif	P14174	IPI00230427	Macrophage migration inhibitory factor	Yes	–	0	0	5.92	0
Prdx1	Q06830	IPI00121788	Peroxiredoxin-1	–	–	0	0	2.80	0
Pkm2	P14618	IPI00845840	Isoform M1 of Pyruvate kinase isozymes M1/M2	–	Yes	0	0	0.80	0
Srpx	P78539	IPI00127815	Isoform 1 of Sushi-repeat-containing protein SRPX	–	Yes	0	0	0.78	0
Prdx5	P30044	IPI00129517	Isoform Mitochondrial of Peroxiredoxin-5, mitochondrial	–	–	0	0	2.80	0
Col4a1	P02462	IPI00109588	Collagen alpha-1(IV) chain	Yes	–	0	0	0.25	0
Rhoc	P08134	IPI00122245	Rho-related GTP-binding protein RhoC	–	Yes	0	0	1.40	0
Mdh2	P40926	IPI00323592	Malate dehydrogenase, mitochondrial	–	Yes	0	0	0.85	0
Lrrc15	Q8TF66	IPI00330681	Leucine-rich repeat-containing protein 15	–	–	0	0	0.48	0
Rpl28	P46779	IPI00222547	60S ribosomal protein L28	–	–	0	0	1.92	0

[a]Proteins not assigned to the Gene Ontology terms GO:0005576 (extracellular region), GO:0005615 (extracellular space), GO:0005886 (plasma membrane) or GO:0009986 (cell surface) are indicated by a dash (–).

SUPPLEMENTAL TABLE S2

Hierarchical clustering analysis of proteins identified in hPSF, ihPSF and HUES1 ECMs (complete datasets)

Cell-derived ECMs were isolated and analysed by MS as described in "Experimental Procedures." Output of hierarchical clustering analysis is represented in Figure 4.

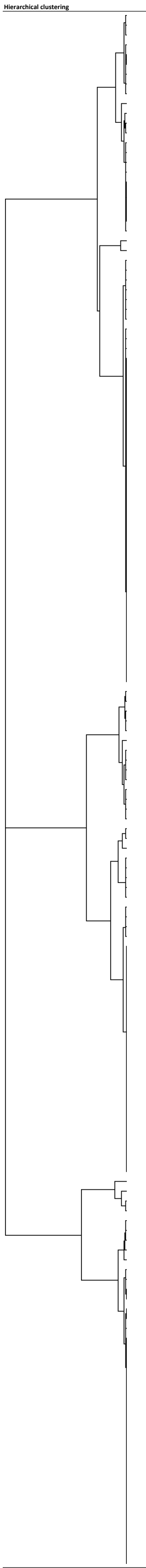

Hierarchical clustering	Human gene symbol	Human UniProt Accession	IPI Accession	Protein name	Extracellular?[a]	Cell surface?[a]	Mean normalised spectrum count (% total spectra) ×10^3 hPSF	ihPSF	HUES1
	TUBB	P07437	IPI00645452	Tubulin, beta	–	–	19.40	5.93	0
	ACTA1	P68133	IPI00021428	Actin, alpha skeletal muscle	–	–	108.59	38.27	0
	EEF1A1	P68104	IPI00396485	Elongation factor 1-alpha 1	–	–	15.59	5.26	0
	KRT2	P35908	IPI00021304	Keratin, type II cytoskeletal 2 epidermal	–	–	45.57	24.36	0
	A2M	P01023	IPI00478003	Alpha-2-macroglobulin	Yes	–	2.24	1.21	0
	RPS27A	P62979	IPI00179330	ubiquitin and ribosomal protein S27a precursor	–	–	6.00	3.25	0
	ATP5B	P06576	IPI00303476	ATP synthase subunit beta, mitochondrial	–	Yes	5.18	2.69	0
	RAB1A	P62820	IPI00005719	Isoform 1 of Ras-related protein Rab-1A	–	–	5.87	2.86	0
	STOM	P27105	IPI00219682	Erythrocyte band 7 integral membrane protein	–	Yes	12.82	5.48	0
	KRT9	P35527	IPI00019359	Keratin, type I cytoskeletal 9	–	–	70.89	43.73	19.30
	KRT1	P04264	IPI00220327	Keratin, type II cytoskeletal 1	–	Yes	106.71	70.53	13.92
	NT5E	P21589	IPI00009456	5'-nucleotidase	–	Yes	16.91	9.86	0
	PHB2	Q99623	IPI00027252	Prohibitin-2	–	–	4.09	2.66	0
	KRT10	P13645	IPI00009865	Keratin, type I cytoskeletal 10	–	–	73.39	54.37	0
	VTN	P04004	IPI00298971	Vitronectin	Yes	–	11.99	8.52	0
	MYL6	P60660	IPI00335168	Isoform Non-muscle of Myosin light polypeptide 6	–	–	44.76	38.25	0
	GDF15	Q99988	IPI00306543	Growth/differentiation factor 15	Yes	–	48.45	38.47	0
	HIST1H2BL	Q99880	IPI00018534	Histone H2B type 1-L	–	–	7.71	6.27	0
	HIST1H4A	P62805	IPI00453473	Histone H4	–	–	9.81	7.97	0
	S100A6	P06703	IPI00027463	Protein S100-A6	–	Yes	13.49	10.96	0
	ARF4	P18085	IPI00215918	ADP-ribosylation factor 4	–	–	5.14	4.17	0
	ATP5O	P48047	IPI00007611	ATP synthase subunit O, mitochondrial	–	Yes	3.52	2.86	0
	GSN	P06396	IPI00026314	Isoform 1 of Gelsolin	Yes	–	0.82	0.68	0
	ACTB	P60709	IPI00021439	Actin, cytoplasmic 1	–	–	157.69	61.40	110.21
	HSP90B1	P14625	IPI00027230	Endoplasmin	–	Yes	10.00	1.19	4.08
	TUBA1B	P68363	IPI00792677	cDNA FLJ60097, highly similar to Tubulin alpha-ubiquitous chain	–	–	20.43	4.76	0
	MYH9	P35579	IPI00019502	Isoform 1 of Myosin-9	–	Yes	30.89	6.18	0
	S100A11	P31949	IPI00013895	Protein S100-A11	–	Yes	28.27	5.48	0
	ANXA1	P04083	IPI00218918	Annexin A1	Yes	–	17.22	2.81	0
	ATP5A1	P25705	IPI00440493	ATP synthase subunit alpha, mitochondrial	–	Yes	7.45	1.10	0
	HSPA5	P11021	IPI00003362	HSPA5 protein	–	Yes	7.45	0.91	0
	SERPINH1	P50454	IPI00032140	Serpin H1	–	–	7.63	0.95	0
	FLNA	P21333	IPI00302592	Isoform 2 of Filamin-A	–	Yes	3.32	0.21	0
	ANXA2	P07355	IPI00418169	Isoform 2 of Annexin A2	Yes	–	16.36	1.10	0
	PRDX1	Q06830	IPI00000874	Peroxiredoxin-1	–	–	22.01	1.99	0
	GNAT2	P19087	IPI00217269	Guanine nucleotide-binding protein G(t) subunit alpha-2	–	Yes	2.66	0	0
	PHB	P35232	IPI00017334	Prohibitin	–	Yes	7.20	0	0
	CKAP4	Q07065	IPI00141318	Isoform 1 of Cytoskeleton-associated protein 4	–	Yes	9.36	0	0
	HSPB1	P04792	IPI00025512	Heat shock protein beta-1	–	Yes	3.52	0	0
	ACO2	Q99798	IPI00017855	Aconitate hydratase, mitochondrial	–	–	0.83	0	0
	RPN1	P04843	IPI00025874	Dolichyl-diphosphooligosaccharide--protein glycosyltransferase subunit 1 precursor	–	–	0.74	0	0
	TAGLN	Q01995	IPI00216138	Transgelin	–	–	14.68	0	0
	CALD1	Q05682	IPI00014516	Isoform 1 of Caldesmon	–	Yes	1.53	0	0
	KRT5	P13647	IPI00009867	Keratin, type II cytoskeletal 5	–	–	1.14	0	0
	ALDOA	P04075	IPI00465439	Fructose-bisphosphate aldolase A	Yes	–	4.55	0	0
	ITGAV	P06756	IPI00027505	Isoform 1 of Integrin alpha-V	–	Yes	2.31	0	0
	HSPA9	P38646	IPI00007765	Stress-70 protein, mitochondrial	–	Yes	3.01	0	0
	LMNA	P02545	IPI00021405	Isoform A of Lamin-A/C	–	–	11.45	0	0
	ACTN1	P12814	IPI00013508	Alpha-actinin-1	–	Yes	5.72	0	0
	P4HB	P07237	IPI00010796	Protein disulfide-isomerase	Yes	–	3.14	0	0
	ANXA5	P08758	IPI00329801	Annexin A5	–	Yes	10.49	0	0
	RPL23A	O14950	IPI00033494	Myosin regulatory light chain 12B	–	–	7.59	0	0
	STOML2	Q9UJZ1	IPI00334190	Stomatin-like protein 2	–	Yes	1.38	0	0
	PCBP1	Q15365	IPI00016610	Poly(rC)-binding protein 1	–	–	1.46	0	0
	TIMP2	P16035	IPI00027166	Metalloproteinase inhibitor 2	Yes	–	2.25	0	0
	PPIB	P23284	IPI00646304	Peptidyl-prolyl cis-trans isomerase B	–	–	4.50	0	0
	HSP90AB1	P08238	IPI00414676	Heat shock protein HSP 90-beta	–	–	1.71	0	0
	MMP2	P08253	IPI00027780	72 kDa type IV collagenase	Yes	–	0.96	0	0
	MYH10	P35580	IPI00397526	Isoform 1 of Myosin-10	–	Yes	4.01	0	0
	TMED10	P49755	IPI00028055	Transmembrane emp24 domain-containing protein 10	–	–	2.16	0	0
	PTRF	Q6NZI2	IPI00176903	Isoform 1 of Polymerase I and transcript release factor	–	Yes	1.26	0	0
	ANPEP	P15144	IPI00221224	Aminopeptidase N	–	Yes	0.64	0	0
	MYO1C	O00159	IPI00010418	Isoform 2 of Myosin-Ic	–	Yes	1.89	0	0
	FLNC	Q14315	IPI00178352	Isoform 1 of Filamin-C	–	Yes	0.74	0	0
	CLTC	Q00610	IPI00024067	Isoform 1 of Clathrin heavy chain 1	–	Yes	0.42	0	0
	CANX	P27824	IPI00020984	cDNA FLJ55574, highly similar to Calnexin	–	–	0.75	0	0
	RPL11	P62913	IPI00376798	Isoform 1 of 60S ribosomal protein L11	–	–	5.40	0	0
	RAB5C	P51148	IPI00016339	Ras-related protein Rab-5C	–	Yes	2.35	0	0
	MYOF	Q9NZM1	IPI00021048	Isoform 1 of Myoferlin	–	Yes	0.23	0	0
	VIM	P08670	IPI00418471	Vimentin	–	Yes	140.53	152.63	0
	TGFBI	Q15582	IPI00018219	Transforming growth factor-beta-induced protein ig-h3	Yes	–	14.32	16.56	0
	SERPINE1	P05121	IPI00007118	Plasminogen activator inhibitor 1	Yes	–	3.94	3.90	0
	HIST1H2AB	P04908	IPI00026272	Histone H2A type 1-B/E	–	–	15.42	14.09	0
	VDAC2	P45880	IPI00024145	Isoform 2 of Voltage-dependent anion-selective channel protein 2	–	–	2.36	2.19	0
	TNC	P24821	IPI00220213	Isoform 4 of Tenascin	Yes	–	12.34	17.21	4.22
	HBA1	P69905	IPI00410714	Hemoglobin subunit alpha	–	–	61.88	88.15	0
	SULF1	Q8IWU6	IPI00293203	Extracellular sulfatase Sulf-1	Yes	–	1.40	2.03	0
	HTRA1	Q92743	IPI00003176	Serine protease HTRA1	Yes	–	71.48	95.00	0
	AFP	P02771	IPI00022443	Alpha-fetoprotein	Yes	–	1.03	1.27	0
	TGM2	P21980	IPI00294578	Isoform 1 of Protein-glutamine gamma-glutamyltransferase 2	Yes	–	10.21	19.16	0
	RPS16	P62249	IPI00221092	40S ribosomal protein S16	–	–	6.75	12.32	0
	RPS25	P62851	IPI00012750	40S ribosomal protein S25	–	–	15.31	25.58	0
	RPS3	P23396	IPI00011253	40S ribosomal protein S3	–	–	2.00	3.25	0
	EMILIN1	Q9Y6C2	IPI00013079	EMILIN-1	Yes	–	24.86	49.66	13.21
	THBS1	P07996	IPI00296099	Thrombospondin-1	Yes	–	1.79	4.93	1.25
	RPS18	P62269	IPI00013296	40S ribosomal protein S18	–	–	4.50	22.32	5.95
	CYR61	O00622	IPI00299219	Protein CYR61	Yes	–	21.55	46.98	2.55
	WNT5A	P41221	IPI00013178	Isoform 2 of Protein Wnt-5a	–	–	7.78	16.22	0
	TFPI2	P48307	IPI00009198	Tissue factor pathway inhibitor 2	Yes	–	10.51	22.46	0
	RAC1	P63000	IPI00010271	Isoform A of Ras-related C3 botulinum toxin substrate 1	–	Yes	2.57	5.91	0
	AHSG	P02765	IPI00022431	cDNA FLJ55606, highly similar to Alpha-2-HS-glycoprotein	Yes	–	1.15	3.26	0
	COL6A3	P12111	IPI00022200	Isoform 1 of Collagen alpha-3(VI) chain	Yes	–	1.25	8.41	0
	COL6A1	P12109	IPI00291136	Collagen alpha-1(VI) chain	Yes	–	0.74	5.23	0
	RPS19	P39019	IPI00215780	40S ribosomal protein S19	–	–	3.37	20.09	0
	COL6A2	P12110	IPI00304840	Isoform 2C2 of Collagen alpha-2(VI) chain	Yes	–	0.98	4.49	0
	RPS13	P62277	IPI00221089	40S ribosomal protein S13	–	–	0	7.73	0
	RPS4X	P62701	IPI00217030	40S ribosomal protein S4, X isoform	–	–	0	3.90	0
	RPS17	P08708	IPI00221093	40S ribosomal protein S17	–	–	0	7.76	0
	RPS29	P62273	IPI00182289	40S ribosomal protein S29	–	–	0	9.39	0
	FLOT1	O75955	IPI00027438	Flotillin-1	–	Yes	0	2.49	0
	EFEMP2	O95967	IPI00296058	EGF-containing fibulin-like extracellular matrix protein 2	Yes	–	0	2.68	0
	CD55	P08174	IPI00152418	Decay-accelerating factor splicing variant 4	–	Yes	0	3.13	0
	MVP	Q14764	IPI00000105	Major vault protein	–	–	0	1.48	0
	IGFBP5	P24593	IPI00029236	Insulin-like growth factor-binding protein 5	Yes	–	0	4.71	0
	CPZ	Q66K79	IPI00179185	Isoform 2 of Carboxypeptidase Z	Yes	–	0	2.40	0
	KIAA1199	Q8WUJ3	IPI00376689	Isoform 1 of Protein KIAA1199	–	–	0	1.72	0
	SERPINF1	P36955	IPI00006114	Pigment epithelium-derived factor	Yes	–	0	3.97	0
	RPS3A	P61247	IPI00419880	40S ribosomal protein S3a	–	–	0	11.20	0
	RPL31	P62899	IPI00026302	60S ribosomal protein L31	–	–	0	18.80	0
	SERPINE2	P07093	IPI00914848	Isoform 2 of Glia-derived nexin	Yes	–	0	1.00	0
	RPS11	P62280	IPI00025091	40S ribosomal protein S11	–	–	0	4.87	0
	RPL9	P32969	IPI00031691	60S ribosomal protein L9	–	–	0	1.99	0
	C3	P01024	IPI00783987	Complement C3 (Fragment)	Yes	–	0	0.31	0
	CTHRC1	Q96CG8	IPI00060423	Isoform 1 of Collagen triple helix repeat-containing protein 1	Yes	–	0	2.25	0
	GREM1	O60565	IPI00298476	Isoform 1 of Gremlin-1	Yes	–	0	4.18	0
	CD44	P16070	IPI00297160	Isoform 12 of CD44 antigen	–	Yes	0	2.25	0
	RPL23A	P62750	IPI00021266	60S ribosomal protein L23a	–	–	0	11.37	0
	COL12A1	Q99715	IPI00302944	Isoform 4 of Collagen alpha-1(XII) chain	Yes	–	0	0.47	0
	FLOT2	Q14254	IPI00789008	Flotillin-2	–	Yes	0	3.11	0
	RPS14	P62263	IPI00026271	40S ribosomal protein S14	–	–	15.51	33.80	30.14
	FN1	Q14328	IPI00845263	fibronectin 1 isoform 2 preproprotein	Yes	–	2.34	57.18	81.93
	VCAN	P13611	IPI00215628	Isoform V1 of Versican core protein	Yes	–	0.94	1.71	3.44
	ADAMTS4	O75173	IPI00307276	A disintegrin and metalloproteinase with thrombospondin motifs 4	Yes	–	1.18	2.03	4.76
	HSPG2	P98160	IPI00024284	Basement membrane-specific heparan sulfate proteoglycan core protein	Yes	–	0	4.16	15.66
	NID1	P14543	IPI00026944	Isoform 1 of Nidogen-1	Yes	–	0	0.64	2.59
	COL4A1	P02462	IPI00743696	Isoform 1 of Collagen alpha-1(IV) chain	Yes	–	0	1.63	5.52
	PKM2	P14618	IPI00220644	Isoform M1 of Pyruvate kinase isozymes M1/M2	–	Yes	0	1.76	4.62
	GAPDH	P04406	IPI00219018	Glyceraldehyde-3-phosphate dehydrogenase	–	–	1.50	2.43	10.42
	COL1A2	P08123	IPI00304962	Collagen alpha-2(I) chain	Yes	–	0	3.06	18.10
	COL1A1	P02452	IPI00297646	Collagen alpha-1(I) chain	Yes	–	1.02	7.36	54.25
	LAMA5	O15230	IPI00783665	Laminin subunit alpha-5	Yes	–	0	0.22	1.82
	FBN1	P35555	IPI00328113	Fibrillin-1	Yes	–	0	0.37	3.09
	COL4A2	P08572	IPI00306322	Collagen alpha-2(IV) chain	Yes	–	0	1.04	18.45
	LAMB1	P07942	IPI00013976	Laminin subunit beta-1	Yes	–	0	0.30	5.29
	LAMC1	P11047	IPI00298281	Laminin subunit gamma-1	Yes	–	0	0.33	10.81
	LAMA1	P25391	IPI00375294	Laminin subunit alpha-1	Yes	–	0	0	1.97
	APOE	P02649	IPI00021842	Apolipoprotein E	Yes	–	0	0	214.69
	TUBB6	Q9BUF5	IPI00930130	cDNA FLJ11352 fis, clone HEMBA1000020, highly similar to Tubulin beta-2C chain	–	–	0	0	5.95
	LEFTY2	O00292	IPI00010893	Left-right determination factor 2	Yes	–	0	0	16.68
	HNRNPM	P52272	IPI00171903	Isoform 1 of Heterogeneous nuclear ribonucleoprotein M	–	Yes	0	0	15.90
	PHGDH	O43175	IPI00011200	D-3-phosphoglycerate dehydrogenase	–	–	0	0	1.88
	EMILIN2	Q9BXX0	IPI00012510	EMILIN-2	Yes	–	0	0	1.39
	KRT8	P05787	IPI00554648	Keratin, type II cytoskeletal 8	–	–	0	0	11.67
	AGRN	O00468	IPI00374563	Agrin	Yes	–	0	0	10.04
	EGFL7	Q9UHF1	IPI00383960	Epidermal growth factor-like protein 7	Yes	–	0	0	12.50
	EMID2	Q96A83	IPI00374096	Isoform 2 of Collagen alpha-1(XXVI) chain	Yes	–	0	0	8.34
	KRT18	P05783	IPI00554788	Keratin, type I cytoskeletal 18	–	–	0	0	19.56
	FBLN2	P98095	IPI00023824	cDNA FLJ56912, highly similar to Fibulin-2	Yes	–	0	0	6.13
	COL2A1	P02458	IPI00186460	Isoform 1 of Collagen alpha-1(II) chain	Yes	–	0	0	1.20
	FGFBP3	Q8TAT2	IPI00301201	Fibroblast growth factor-binding protein 3	Yes	–	0	0	5.74
	COL18A1	P39060	IPI00022822	Isoform 2 of Collagen alpha-1(XVIII) chain	Yes	–	0	0	5.83
	CLU	P10909	IPI00291262	Isoform 1 of Clusterin	Yes	–	0	0	43.13
	FBN2	P35556	IPI00019439	Isoform 1 of Fibrillin-2	Yes	–	0	0	10.97
	NID2	Q14112	IPI00028908	Isoform 1 of Nidogen-2	Yes	–	0	0	1.06
	MTHFD1	P11586	IPI00218342	C-1-tetrahydrofolate synthase, cytoplasmic	–	–	0	0	1.05
	EPRS	P07814	IPI00013452	Bifunctional aminoacyl-tRNA synthetase	–	–	0	0	0.63
	DHX9	Q08211	IPI00844578	ATP-dependent RNA helicase A	–	–	0	0	1.14
	CCT8	P50990	IPI00302925	59 kDa protein	–	–	0	0	1.82
	EEF2	P13639	IPI00186290	Elongation factor 2	–	–	0	0	1.69

[a]Proteins not assigned to the Gene Ontology terms GO:0005576 (extracellular region), GO:0005615 (extracellular space), GO:0005886 (plasma membrane) or GO:0009986 (cell surface) are indicated by a dash (–).

SUPPLEMENTAL TABLE S3

Functional enrichment analysis of clusters of proteins identified in CD1 and MF1×CD1 ECMs.

Clusters of proteins identified by hierarchical clustering analysis (Figure 3) were subjected to Gene Ontology enrichment analysis (as described in "Experimental Procedures").

Cluster[a]	Category[b]	Term	Count[c]	Genes	Fold enrichment	Corrected *P* value[b]
1	GOTERM_BP_FAT	–				
2	GOTERM_BP_FAT	GO:0022610~biological adhesion	11	VCAM1, CD44, NPNT, TGFBI, COL6A3, COL6A2, COL6A1, COL12A1, NID2, EMILIN2, ITGB1	7.32	1.61E-04
2	GOTERM_BP_FAT	GO:0030198~extracellular matrix organization	6	ADAMTSL4, TGFBI, COL1A2, COL6A2, COL12A1, COL1A1	26.91	3.05E-04
2	GOTERM_BP_FAT	GO:0007155~cell adhesion	11	VCAM1, CD44, NPNT, TGFBI, COL6A3, COL6A2, COL6A1, COL12A1, NID2, EMILIN2, ITGB1	7.33	3.19E-04
2	GOTERM_BP_FAT	GO:0043062~extracellular structure organization	6	ADAMTSL4, TGFBI, COL1A2, COL6A2, COL12A1, COL1A1	17.17	2.06E-03
2	GOTERM_BP_FAT	GO:0001501~skeletal system development	6	FBN1, COL1A2, COL12A1, GNAS, COL1A1, MMP14	8.77	3.84E-02
3	GOTERM_BP_FAT	GO:0042060~wound healing	7	GSN, FBLN5, SERPINE1, LOX, TPM1, TGFB2, FN1	21.56	3.57E-04
3	GOTERM_BP_FAT	GO:0009611~response to wounding	9	GSN, FBLN5, SERPINE1, KRT1, LOX, THBS1, TPM1, TGFB2, FN1	9.99	4.09E-04
3	GOTERM_BP_FAT	GO:0030198~extracellular matrix organization	5	FBLN5, POSTN, LOX, CYR61, TGFB2	28.28	5.68E-03
3	GOTERM_BP_FAT	GO:0001944~vasculature development	6	LOX, MYH9, THBS1, CYR61, THY1, TGFB2	14.06	6.76E-03
3	GOTERM_BP_FAT	GO:0001568~blood vessel development	6	LOX, MYH9, THBS1, CYR61, THY1, TGFB2	14.40	7.52E-03
3	GOTERM_BP_FAT	GO:0001525~angiogenesis	5	MYH9, THBS1, CYR61, THY1, TGFB2	19.87	7.54E-03
3	GOTERM_BP_FAT	GO:0022610~biological adhesion	8	FBLN5, MGP, POSTN, MYH9, THBS1, CYR61, THY1, FN1	6.71	8.23E-03
3	GOTERM_BP_FAT	GO:0030155~regulation of cell adhesion	5	GSN, THBS1, TPM1, CYR61, TGFB2	21.47	8.37E-03
3	GOTERM_BP_FAT	GO:0043062~extracellular structure organization	5	FBLN5, POSTN, LOX, CYR61, TGFB2	18.04	8.96E-03
3	GOTERM_BP_FAT	GO:0007155~cell adhesion	8	FBLN5, MGP, POSTN, MYH9, THBS1, CYR61, THY1, FN1	6.72	9.31E-03
3	GOTERM_BP_FAT	GO:0045785~positive regulation of cell adhesion	4	THBS1, TPM1, CYR61, TGFB2	39.21	9.45E-03
3	GOTERM_BP_FAT	GO:0010035~response to inorganic substance	5	GSN, SERPINE1, MGP, THBS1, TPM1	14.35	1.97E-02
3	GOTERM_BP_FAT	GO:0048514~blood vessel morphogenesis	5	MYH9, THBS1, CYR61, THY1, TGFB2	13.94	2.03E-02
3	GOTERM_BP_FAT	GO:0030048~actin filament-based movement	3	ACTC1, MYH9, TPM1	76.72	3.47E-02
3	GOTERM_BP_FAT	GO:0006928~cell motion	6	VIM, MYH9, THBS1, TPM1, TGFB2, FN1	7.43	4.05E-02
3	GOTERM_BP_FAT	GO:0010033~response to organic substance	7	ACTC1, GSN, MGP, LOX, THBS1, CYR61, TGFB2	5.71	4.14E-02
4	GOTERM_BP_FAT	–				
5	GOTERM_BP_FAT	GO:0034220~ion transmembrane transport	4	ATP5B, ATP6V0A1, ATP5O, ATP5A1	92.03	1.21E-03
5	GOTERM_BP_FAT	GO:0006818~hydrogen transport	4	ATP5B, ATP6V0A1, ATP5O, ATP5A1	71.58	1.30E-03
5	GOTERM_BP_FAT	GO:0015985~energy coupled proton transport, down electrochemical gradient	4	ATP5B, ATP6V0A1, ATP5O, ATP5A1	112.73	1.31E-03
5	GOTERM_BP_FAT	GO:0015986~ATP synthesis coupled proton transport	4	ATP5B, ATP6V0A1, ATP5O, ATP5A1	112.73	1.31E-03
5	GOTERM_BP_FAT	GO:0015992~proton transport	4	ATP5B, ATP6V0A1, ATP5O, ATP5A1	73.92	1.57E-03
5	GOTERM_BP_FAT	GO:0006200~ATP catabolic process	3	ATP5B, ATP5O, ATP5A1	211.37	2.41E-03
5	GOTERM_BP_FAT	GO:0009146~purine nucleoside triphosphate catabolic process	3	ATP5B, ATP5O, ATP5A1	161.05	2.63E-03
5	GOTERM_BP_FAT	GO:0009260~ribonucleotide biosynthetic process	4	ATP5B, ATP6V0A1, ATP5O, ATP5A1	36.37	2.63E-03
5	GOTERM_BP_FAT	GO:0009203~ribonucleoside triphosphate catabolic process	3	ATP5B, ATP5O, ATP5A1	178.00	2.63E-03
5	GOTERM_BP_FAT	GO:0009207~purine ribonucleoside triphosphate catabolic process	3	ATP5B, ATP5O, ATP5A1	178.00	2.63E-03
5	GOTERM_BP_FAT	GO:0046034~ATP metabolic process	4	ATP5B, ATP6V0A1, ATP5O, ATP5A1	42.95	2.67E-03
5	GOTERM_BP_FAT	GO:0009144~purine nucleoside triphosphate metabolic process	4	ATP5B, ATP6V0A1, ATP5O, ATP5A1	36.96	2.68E-03
5	GOTERM_BP_FAT	GO:0009141~nucleoside triphosphate metabolic process	4	ATP5B, ATP6V0A1, ATP5O, ATP5A1	34.42	2.73E-03
5	GOTERM_BP_FAT	GO:0009142~nucleoside triphosphate biosynthetic process	4	ATP5B, ATP6V0A1, ATP5O, ATP5A1	44.21	2.76E-03
5	GOTERM_BP_FAT	GO:0009143~nucleoside triphosphate catabolic process	3	ATP5B, ATP5O, ATP5A1	140.92	2.76E-03
5	GOTERM_BP_FAT	GO:0009154~purine ribonucleotide catabolic process	3	ATP5B, ATP5O, ATP5A1	147.04	2.81E-03
5	GOTERM_BP_FAT	GO:0009199~ribonucleoside triphosphate metabolic process	4	ATP5B, ATP6V0A1, ATP5O, ATP5A1	38.21	2.84E-03
5	GOTERM_BP_FAT	GO:0009150~purine ribonucleotide metabolic process	4	ATP5B, ATP6V0A1, ATP5O, ATP5A1	32.68	2.85E-03
5	GOTERM_BP_FAT	GO:0009261~ribonucleotide catabolic process	3	ATP5B, ATP5O, ATP5A1	135.28	2.85E-03
5	GOTERM_BP_FAT	GO:0009201~ribonucleoside triphosphate biosynthetic process	4	ATP5B, ATP6V0A1, ATP5O, ATP5A1	45.55	2.88E-03
5	GOTERM_BP_FAT	GO:0009145~purine nucleoside triphosphate biosynthetic process	4	ATP5B, ATP6V0A1, ATP5O, ATP5A1	45.55	2.88E-03
5	GOTERM_BP_FAT	GO:0006164~purine nucleotide biosynthetic process	4	ATP5B, ATP6V0A1, ATP5O, ATP5A1	30.47	2.90E-03
5	GOTERM_BP_FAT	GO:0006754~ATP biosynthetic process	4	ATP5B, ATP6V0A1, ATP5O, ATP5A1	50.67	2.93E-03
5	GOTERM_BP_FAT	GO:0009259~ribonucleotide metabolic process	4	ATP5B, ATP6V0A1, ATP5O, ATP5A1	30.68	2.97E-03
5	GOTERM_BP_FAT	GO:0009205~purine ribonucleoside triphosphate metabolic process	4	ATP5B, ATP6V0A1, ATP5O, ATP5A1	38.54	3.02E-03
5	GOTERM_BP_FAT	GO:0009152~purine ribonucleotide biosynthetic process	4	ATP5B, ATP6V0A1, ATP5O, ATP5A1	38.54	3.02E-03
5	GOTERM_BP_FAT	GO:0006119~oxidative phosphorylation	4	ATP5B, ATP6V0A1, ATP5O, ATP5A1	46.01	3.26E-03
5	GOTERM_BP_FAT	GO:0009206~purine ribonucleoside triphosphate biosynthetic process	4	ATP5B, ATP6V0A1, ATP5O, ATP5A1	46.01	3.26E-03
5	GOTERM_BP_FAT	GO:0006195~purine nucleotide catabolic process	3	ATP5B, ATP5O, ATP5A1	102.48	4.37E-03
5	GOTERM_BP_FAT	GO:0009165~nucleotide biosynthetic process	4	ATP5B, ATP6V0A1, ATP5O, ATP5A1	24.24	5.22E-03
5	GOTERM_BP_FAT	GO:0006163~purine nucleotide metabolic process	4	ATP5B, ATP6V0A1, ATP5O, ATP5A1	24.24	5.22E-03
5	GOTERM_BP_FAT	GO:0034404~nucleobase, nucleoside and nucleotide biosynthetic process	4	ATP5B, ATP6V0A1, ATP5O, ATP5A1	23.36	5.59E-03
5	GOTERM_BP_FAT	GO:0034654~nucleobase, nucleoside, nucleotide and nucleic acid biosynthetic process	4	ATP5B, ATP6V0A1, ATP5O, ATP5A1	23.36	5.59E-03
5	GOTERM_BP_FAT	GO:0009166~nucleotide catabolic process	3	ATP5B, ATP5O, ATP5A1	66.31	9.29E-03
5	GOTERM_BP_FAT	GO:0034656~nucleobase, nucleoside and nucleotide catabolic process	3	ATP5B, ATP5O, ATP5A1	58.31	1.16E-02
5	GOTERM_BP_FAT	GO:0034655~nucleobase, nucleoside, nucleotide and nucleic acid catabolic process	3	ATP5B, ATP5O, ATP5A1	58.31	1.16E-02
5	GOTERM_BP_FAT	GO:0044270~nitrogen compound catabolic process	3	ATP5B, ATP5O, ATP5A1	50.48	1.49E-02
5	GOTERM_BP_FAT	GO:0046700~heterocycle catabolic process	3	ATP5B, ATP5O, ATP5A1	43.92	1.89E-02
5	GOTERM_BP_FAT	GO:0006091~generation of precursor metabolites and energy	4	ATP5B, ATP6V0A1, ATP5O, ATP5A1	14.41	1.89E-02
5	GOTERM_BP_FAT	GO:0015672~monovalent inorganic cation transport	4	ATP5B, ATP6V0A1, ATP5O, ATP5A1	14.18	1.92E-02
5	GOTERM_BP_FAT	GO:0044271~nitrogen compound biosynthetic process	4	ATP5B, ATP6V0A1, ATP5O, ATP5A1	13.87	1.98E-02
5	GOTERM_BP_FAT	GO:0010941~regulation of cell death	5	PHB, HSPB1, HSPD1, TIMP3, HSPA9	6.92	2.83E-02
5	GOTERM_BP_FAT	GO:0042981~regulation of apoptosis	5	PHB, HSPB1, HSPD1, TIMP3, HSPA9	7.01	2.85E-02
5	GOTERM_BP_FAT	GO:0043067~regulation of programmed cell death	5	PHB, HSPB1, HSPD1, TIMP3, HSPA9	6.94	2.87E-02
5	GOTERM_BP_FAT	GO:0009136~purine nucleoside diphosphate biosynthetic process	2	ATP5B, ATP5A1	450.93	3.63E-02
5	GOTERM_BP_FAT	GO:0006172~ADP biosynthetic process	2	ATP5B, ATP5A1	450.93	3.63E-02
5	GOTERM_BP_FAT	GO:0009180~purine ribonucleoside diphosphate biosynthetic process	2	ATP5B, ATP5A1	450.93	3.63E-02
5	GOTERM_BP_FAT	GO:0009188~ribonucleoside diphosphate biosynthetic process	2	ATP5B, ATP5A1	450.93	3.63E-02
5	GOTERM_BP_FAT	GO:0046031~ADP metabolic process	2	ATP5B, ATP5A1	375.78	4.22E-02
5	GOTERM_BP_FAT	GO:0009133~nucleoside diphosphate biosynthetic process	2	ATP5B, ATP5A1	322.10	4.79E-02
6	GOTERM_BP_FAT	GO:0030198~extracellular matrix organization	6	COL4A2, NID1, VWA1, COL5A2, COL5A1, ANXA2	17.74	1.49E-02

[a]Clusters numbered from top to bottom, as displayed in Figure 3.

[b]Gene Ontology enrichment analysis was performed using the biological process domain against a *Homo sapiens* background.

[c]Number of proteins identified by MS associated with each term.

[d]Benjamini-Hochberg-corrected *P* values ≤0.05 using the modified Fisher's exact test implemented in DAVID were considered significant.

SUPPLEMENTAL TABLE S4

Functional enrichment analysis of clusters of proteins identified in hPSF, ihPSF and HUES1 ECMs.

Clusters of proteins identified by hierarchical clustering analysis (Figure 4) were subjected to Gene Ontology enrichment analysis (as described in "Experimental Procedures").

Cluster[a]	Category[b]	Term	Count[c]	Genes	Fold enrichment	Corrected *P* value[b]
1	GOTERM_BP_FAT	GO:0009261~ribonucleotide catabolic process	3	ATP5B, ATP5O, NT5E	162.34	1.61E-02
1	GOTERM_BP_FAT	GO:0006195~purine nucleotide catabolic process	3	ATP5B, ATP5O, NT5E	122.98	1.88E-02
1	GOTERM_BP_FAT	GO:0009154~purine ribonucleotide catabolic process	3	ATP5B, ATP5O, NT5E	176.45	2.70E-02
1	GOTERM_BP_FAT	GO:0009166~nucleotide catabolic process	3	ATP5B, ATP5O, NT5E	79.58	3.36E-02
1	GOTERM_BP_FAT	GO:0034655~nucleobase, nucleoside, nucleotide and nucleic acid catabolic process	3	ATP5B, ATP5O, NT5E	69.97	3.47E-02
1	GOTERM_BP_FAT	GO:0034656~nucleobase, nucleoside and nucleotide catabolic process	3	ATP5B, ATP5O, NT5E	69.97	3.47E-02
1	GOTERM_BP_FAT	GO:0044270~nitrogen compound catabolic process	3	ATP5B, ATP5O, NT5E	60.57	3.85E-02
1	GOTERM_BP_FAT	GO:0046700~heterocycle catabolic process	3	ATP5B, ATP5O, NT5E	52.71	4.34E-02
2	GOTERM_BP_FAT	GO:0030029~actin filament-based process	6	ALDOA, CALD1, ACTN1, MYH9, FLNA, MYH10	12.47	1.52E-02
2	GOTERM_BP_FAT	GO:0030036~actin cytoskeleton organization	6	ALDOA, CALD1, ACTN1, MYH9, FLNA, MYH10	13.30	1.68E-02
2	GOTERM_BP_FAT	GO:0006916~anti-apoptosis	6	HSP90B1, ANXA1, HSPB1, HSPA5, ANXA5, HSPA9	14.59	2.15E-02
2	GOTERM_BP_FAT	GO:0010941~regulation of cell death	8	HSP90B1, PHB, ANXA1, HSPB1, ACTN1, HSPA5, ANXA5, HSPA9	4.92	3.63E-02
2	GOTERM_BP_FAT	GO:0043067~regulation of programmed cell death	8	HSP90B1, PHB, ANXA1, HSPB1, ACTN1, HSPA5, ANXA5, HSPA9	4.94	3.94E-02
2	GOTERM_BP_FAT	GO:0060548~negative regulation of cell death	6	HSP90B1, ANXA1, HSPB1, HSPA5, ANXA5, HSPA9	8.35	4.14E-02
2	GOTERM_BP_FAT	GO:0042981~regulation of apoptosis	8	HSP90B1, PHB, ANXA1, HSPB1, ACTN1, HSPA5, ANXA5, HSPA9	4.99	4.17E-02
2	GOTERM_BP_FAT	GO:0007015~actin filament organization	4	ALDOA, CALD1, ACTN1, FLNA	27.84	4.62E-02
2	GOTERM_BP_FAT	GO:0043069~negative regulation of programmed cell death	6	HSP90B1, ANXA1, HSPB1, HSPA5, ANXA5, HSPA9	8.37	4.76E-02
3	GOTERM_BP_FAT	–				
4	GOTERM_BP_FAT	GO:0022610~biological adhesion	10	CD44, FLOT2, COL6A3, RAC1, COL6A2, COL6A1, COL12A1, THBS1, EMILIN1, CYR61	9.19	6.62E-05
4	GOTERM_BP_FAT	GO:0007155~cell adhesion	10	CD44, FLOT2, COL6A3, RAC1, COL6A2, COL6A1, COL12A1, THBS1, EMILIN1, CYR61	9.20	1.31E-04
4	GOTERM_BP_FAT	GO:0009611~response to wounding	8	CD55, CD44, C3, EFEMP2, RAC1, THBS1, TFPI2, AHSG	9.72	1.16E-03
4	GOTERM_BP_FAT	GO:0030334~regulation of cell migration	5	SERPINE2, RAC1, GREM1, THBS1, IGFBP5	19.06	8.34E-03
4	GOTERM_BP_FAT	GO:0006954~inflammatory response	6	CD55, CD44, C3, RAC1, THBS1, AHSG	11.89	9.18E-03
4	GOTERM_BP_FAT	GO:0032101~regulation of response to external stimulus	5	SERPINF1, C3, GREM1, THBS1, AHSG	20.26	9.87E-03
4	GOTERM_BP_FAT	GO:0051270~regulation of cell motion	5	SERPINE2, RAC1, GREM1, THBS1, IGFBP5	16.69	1.04E-02
4	GOTERM_BP_FAT	GO:0040012~regulation of locomotion	5	SERPINE2, RAC1, GREM1, THBS1, IGFBP5	16.78	1.17E-02
4	GOTERM_BP_FAT	GO:0030198~extracellular matrix organization	4	COL6A2, COL12A1, EMILIN1, CYR61	24.78	2.59E-02
4	GOTERM_BP_FAT	GO:0010811~positive regulation of cell-substrate adhesion	3	THBS1, EMILIN1, CYR61	69.02	3.88E-02
5	GOTERM_BP_FAT	–				
6	GOTERM_BP_FAT	GO:0043062~extracellular structure organization	7	COL18A1, COL4A2, COL1A2, COL2A1, AGRN, LAMC1, COL1A1	27.66	5.20E-05
6	GOTERM_BP_FAT	GO:0030198~extracellular matrix organization	6	COL18A1, COL4A2, COL1A2, COL2A1, LAMC1, COL1A1	37.16	9.53E-05
6	GOTERM_BP_FAT	GO:0030334~regulation of cell migration	6	COL18A1, LAMA1, EGFL7, APOE, LAMA5, LAMB1	22.87	7.04E-04
6	GOTERM_BP_FAT	GO:0051270~regulation of cell motion	6	COL18A1, LAMA1, EGFL7, APOE, LAMA5, LAMB1	20.03	8.08E-04
6	GOTERM_BP_FAT	GO:0040012~regulation of locomotion	6	COL18A1, LAMA1, EGFL7, APOE, LAMA5, LAMB1	20.13	9.85E-04
6	GOTERM_BP_FAT	GO:0001944~vasculature development	6	COL18A1, EGFL7, APOE, LAMA5, COL1A2, COL1A1	15.40	2.06E-03
6	GOTERM_BP_FAT	GO:0001568~blood vessel development	6	COL18A1, EGFL7, APOE, LAMA5, COL1A2, COL1A1	15.78	2.14E-03
6	GOTERM_BP_FAT	GO:0034446~substrate adhesion-dependent cell spreading	3	LAMA5, LAMC1, LAMB1	276.08	2.18E-03
6	GOTERM_BP_FAT	GO:0030155~regulation of cell adhesion	5	LAMA1, FBLN2, LAMA5, EMID2, COL1A1	23.51	2.38E-03
6	GOTERM_BP_FAT	GO:0022610~biological adhesion	8	COL18A1, LAMA1, LAMA5, COL2A1, NID2, LAMC1, LAMB1, EMILIN2	7.35	2.56E-03
6	GOTERM_BP_FAT	GO:0007155~cell adhesion	8	COL18A1, LAMA1, LAMA5, COL2A1, NID2, LAMC1, LAMB1, EMILIN2	7.36	2.85E-03
6	GOTERM_BP_FAT	GO:0042476~odontogenesis	4	LAMA5, COL1A2, COL1A1, LAMB1	47.72	3.01E-03
6	GOTERM_BP_FAT	GO:0000902~cell morphogenesis	6	COL18A1, LAMA1, LAMA5, CLU, LAMC1, LAMB1	10.86	5.82E-03
6	GOTERM_BP_FAT	GO:0032989~cellular component morphogenesis	6	COL18A1, LAMA1, LAMA5, CLU, LAMC1, LAMB1	9.74	8.99E-03
6	GOTERM_BP_FAT	GO:0031589~cell-substrate adhesion	4	LAMA5, NID2, LAMC1, LAMB1	26.29	1.41E-02
6	GOTERM_BP_FAT	GO:0042127~regulation of cell proliferation	7	COL18A1, LAMA1, APOE, LAMA5, CLU, LAMC1, LAMB1	5.73	2.49E-02
6	GOTERM_BP_FAT	GO:0030199~collagen fibril organization	3	COL1A2, COL2A1, COL1A1	66.64	2.64E-02
6	GOTERM_BP_FAT	GO:0050679~positive regulation of epithelial cell proliferation	3	LAMA1, LAMC1, LAMB1	47.14	4.93E-02
6	GOTERM_BP_FAT	GO:0010810~regulation of cell-substrate adhesion	3	FBLN2, EMID2, COL1A1	42.01	5.84E-02

[a]Clusters numbered from top to bottom, as displayed in Figure 4.

[b]Gene Ontology enrichment analysis was performed using the biological process domain against a *Homo sapiens* background.

[c]Number of proteins identified by MS associated with each term.

[d]Benjamini-Hochberg-corrected *P* values ≤0.05 using the modified Fisher's exact test implemented in DAVID were considered significant.
